# Efficacy and safety of stellate ganglion block for postoperative pain management: a systematic review and meta-analysis of randomized controlled trials

**DOI:** 10.1186/s12871-026-03901-0

**Published:** 2026-05-11

**Authors:** Weiwei Liu, Demei Liu, Shengda Liu

**Affiliations:** 1 Department of Anesthesiology, Weifang People’s Hospital, Weifang, Shandong 261000 China; 2 Department of Pathology, Weifang People’s Hospital, Weifang, Shandong 261000 China; 3 Intensive care unit, Weifang People’s Hospital, Weifang, Shandong 261000 China

**Keywords:** Stellate ganglion block, Postoperative pain management, Meta-analysis, Randomized controlled trials

## Abstract

**Background:**

Stellate ganglion block (SGB) is a form of regional anesthesia employed to reduce postoperative pain management. This meta-analysis assesses its efficacy and safety for postoperative pain control.

**Methods:**

We conducted a comprehensive search of PubMed, Embase, Cochrane Library, and Web of Science covering all records up to September 20,2025 to identify eligible randomized controlled trials (RCTs). Stata 15.0 software was applied for data analysis, and RevMan 5.4 was employed to assess the risk of bias.

**Results:**

A total 33 RCTs comprising 2231 participants were included, SGB may reduce pain at 6[SMD=-1.35, 95%CI(-1.96, -0.73)], 12[SMD=-1.09, 95%CI(-1.83, -0.35)]and 24[SMD=-1.03, 95%CI(-1.56, -0.20)] hours postoperatively, whereas no significant difference was observed at 48 h and postoperative total opioid consumption [SMD = − 0.55, 95% CI (− 1.24, 0.13)] and and ICU stays [MD=-0.37, 95% CI(− 0.95, 0.21)]. It shortened hospital stay [MD = − 1.04, 95% CI (− 1.74, − 0.34)], reduced the incidence of postoperative nausea and vomiting[RR = 0.55, 95% CI (0.40, 0.77)], but the incidence of upper eyelid ptosis was markedly higher than in the control group [RR = 31.67, 95% CI (6.43, 155.90)].

**Conclusions:**

Stellate ganglion block may help alleviate early pain (6, 12, and 24 h) postoperatively, It also shortens hospital and the ICU stays and reduces the incidence of nausea and vomiting but increases the incidence of upper eyelid ptosis. Analysis showed no significant group-to-group differences in other indicators. Given the limitations of the included studies, further high-quality, large-sample randomized controlled trials are needed to confirm and expand upon the efficacy and safety of stellate ganglion block.

**Supplementary Information:**

The online version contains supplementary material available at 10.1186/s12871-026-03901-0.

## Background

In recent years, persistent postoperative pain after most surgical procedures has become common, seriously affecting patients’ recovery and quality of life [1]. Poor postoperative pain control can lead to various serious complications, such as cardiopulmonary events, prolonged hospital stays, development of chronic pain, opioid-related adverse effects, opioid-induced hyperalgesia, and addiction [2]. Adequate postoperative analgesia is therefore essential to prevent acute and chronic complications resulting from uncontrolled postoperative pain [3]. Although multimodal analgesia (such as “opioids + nonsteroidal anti-inflammatory drugs + local anesthetics”) can optimize pain relief and reduce the adverse effects of single agents to some extent, many limitations remain. Opioids can effectively alleviate pain but are frequently linked to adverse reactions including nausea and vomiting, respiratory depression, and constipation [4]. Traditional regional blocks can provide precise analgesia at the surgical site, but their range of action is limited and they cannot improve postoperative systemic inflammatory responses or visceral pain [5]. In addition, some surgical patients may develop chronic postoperative pain, which is closely related to prolonged activation of the sympathetic nervous system; however, current analgesic strategies mainly focus on “acute pain control” and lack early intervention for chronic pain [6]. 

The cervicothoracic (stellate) ganglion is a sympathetic structure with a star-like shape, created by the merging of the inferior cervical and first thoracic sympathetic ganglia. Stellate ganglion block (SGB) is an invasive analgesic technique that involves blocking the pre- and post-ganglionic fibers of the cervical sympathetic trunk, vertebral artery ganglion, inferior cervical sympathetic ganglion, and upper thoracic sympathetic ganglion. This procedure improves circulation, metabolism, and pain arising from sympathetic nervous dysfunction. Its purpose is to relieve acute pain and prevent its transition to chronic pain by interrupting central sensitization loops—an important factor in the development of persistent, intractable pain [7]. Stellate ganglion block has unique advantages. It offers a non-opioid pain management option that can avoid or reduce drug dependence, effectively helping patients reduce or even discontinue related medications and thereby improve quality of life and safety [8]. Ultrasound-guided stellate ganglion block further enhances localization accuracy; without the need for systemic medication, it can achieve a combined “local + systemic” analgesic effect through local blockade alone [9].

Previous meta-analyses [10] have shown that stellate ganglion block is effective in the short term after surgery, however, its limitations lie in the low level of evidence and the limited number of included trials, failing to analyze whether SGB can effectively reduce postoperative opioid drug consumption. It also failed to systematically assess the occurrence of complications related to SGB procedures (such as intravascular injection, hematoma, recurrent laryngeal nerve block, etc.). Dong et al. [11] performed a meta-analysis describing the efficacy of stellate ganglion block for analgesia in cancer surgery, however, its limitations lie in significant clinical heterogeneity and complex sources, flawed study design, and the absence of key outcome data (long-term efficacy, safety).

Because the efficacy and safety of this type of block remain under debate, we conducted a meta-analysis as an alternative option for perioperative regional anesthesia. To evaluate the efficacy and safety of SGB for postoperative pain management by synthesizing evidence from RCTs across surgical procedures, and to explore potential sources of heterogeneity. We sought to examine both early and late postoperative pain relief, assess whether stellate ganglion block can effectively reduce the use of postoperative opioid drugs to help minimize drug dependence and related adverse effects, and present a comprehensive evaluation of its effectiveness in postoperative pain management. We also assessed its impact on hospital stay, ICU stay, and the incidence of common postoperative complications to offer a more complete safety profile for clinical practice.

## Methods

This systematic review and meta-analysis was reported in accordance with the PRISMA guidelines and was prospectively registered in PROSPERO (CRD420251135980).

### Literature search

A search of the Cochrane Library, EMBASE, Web of Science, and PubMed was performed using terms related to stellate ganglion block (SGB, stellate ganglion block) linked with “OR,” and surgery-related terms (general surgery, surgery, general; surgery) also linked with “OR.” The two term sets were then combined with “AND.” No restrictions were placed on publication date, patient age, sex, article type, or language. The search was conducted up to August 13, 2025. Supplementary Table S1 show the full search strategies.

### Inclusion and exclusion criteria

The study included participants meeting the following criteria: (a) patients undergoing surgery; (b) those receiving stellate ganglion block (SGB) as the intervention group (SGB group) compared with those who did not receive a block or received a sham block as placebo treatment (control group); (c) primary outcomes: postoperative pain at various time points (6, 12, 24, and 48 h); secondary outcomes: postoperative opioid consumption, operative time, length of hospital stay, ICU stay, complications, and sleep quality.

The exclusion criteria were: the publications in the form of conference abstracts, reviews, meta-analyses, study protocols, case reports, animal experiments, nonrandomized controlled trials, retrospective studies, and incomplete clinical trials; articles without full-text availability; and articles with no usable data.

### Data extraction

In line with the inclusion and exclusion criteria, two reviewers (WWL and SDL) independently performed the screening, and differences were reconciled by involving a third reviewer (DML). Text, tables, and figures from the original articles were evaluated, and the following data were collected: first author, year of publication, country, patient characteristics, type of surgery, treatment method in the stellate ganglion block group, treatment method in the control group, and outcome measures.

The outcome measures included operative time, postoperative complications, length of hospital stay, ICU stay, pain, postoperative opioid consumption, and sleep quality. Pain outcomes were assessed using either the Visual Analogue Scale (VAS) or the Numerical Rating Scale (NRS), which were considered equivalent [12]. For continuous variables, the mean and the standard deviation of each outcome were extracted. If these data were not reported, the mean was estimated as the median and the standard deviation was estimated from the range and median according to the method presented by Wang et al. [13]. For dichotomous variables, the number of events (incidence) for each outcome was extracted.

### Quality assessment

The quality of the randomized controlled trials included was independently appraised by two reviewers (WWL and SDL) using the Cochrane risk-of-bias tool [14], and any disagreements were resolved by consulting a third reviewer (DML) to achieve consensus. The quality assessment covered seven domains: allocation concealment, incomplete outcome data, blinding of outcome assessors, blinding of participants and personnel, random sequence generation, selective reporting, and other biases. Risk of bias in each domain was classified as low, high, or unclear. All included studies were evaluated separately against these criteria: full compliance was rated “low risk,” partial compliance “unclear risk,” and non-compliance “high risk.”

### GRADE evaluation

The quality of evidence for primary outcome measures was assessed using the Grading of Recommendations Assessment, Development and Evaluation (GRADE) approach. The GRADE system comprehensively evaluates the overall quality of evidence from meta-analyses across five dimensions: study limitations (risk of bias), inconsistency of results, indirectness, imprecision, and publication bias. Evidence from randomized controlled trials was initially rated as “high” quality, with downgrades applied as appropriate based on the aforementioned factors. Final evidence quality was categorized into four levels: high, moderate, low, or very low. Evidence quality assessments were conducted independently by two researchers, with disagreements resolved through discussion and consensus.

### Statistical analysis

All analyses were performed using Stata 15.0 software. For continuous variables, standardized mean differences (SMDs) with 95% confidence intervals (CIs) were used to assess the significance of differences. When the outcomes were reported as continuous data (e.g., pain scores, opioid consumption), the mean difference (MD) was used in place of the SMD, and results are presented with 95% CIs. For dichotomous variables, risk ratios (RRs) with 95% CIs were determined. Forest plots were generated to visually display the effects of interventions, with the I² statistic used to assess heterogeneity across studies.

Heterogeneity was quantified using the I² statistic. When I² exceeded 50%, it was considered substantial, and a random-effects model was employed. In cases where I² was less than 50%, we used a fixed-effect model for analysis. Sensitivity analyses were conducted by sequentially omitting each study to explore potential sources of heterogeneity and determine whether the exclusion of any single study would markedly alter the results.

Subgroup analysis was performed based on different factors such as surgical type, surgical approach, block level, ultrasound guidance, and block timing. In this analysis, we stratified the studies into relevant groups to explore how each subgroup may impact the overall treatment effect. Subgroup analysis allows for a deeper understanding of the present in the results and to identify specific factors that contribute to variations in outcomes. For each subgroup, heterogeneity was assessed, and statistical significance was determined by comparing the MD (Mean Difference) or SMD with corresponding 95% confidence intervals.

Meta-regression analysis was used to explore the effect of potential study-level covariates (e.g., surgical type, surgical approach, block timing, ultrasound guidance, year of study) on the overall treatment effects. Regression coefficients (Coef) and corresponding p-values were reported for each covariate. This analysis helps identify potential sources of heterogeneity and the impact of these variables on the treatment outcomes. A p-value of < 0.05 was considered statistically significant.

Publication bias was assessed using Egger’s test, with p-values of < 0.05 indicating the presence of publication bias. For each outcome, funnel plots were also constructed to visually inspect the symmetry of the distribution of effect sizes. In the presence of asymmetry, we applied the trim-and-fill method to adjust for potential publication bias. If publication bias was detected, the trim-and-fill method helped to adjust the effect sizes to mitigate bias. The funnel plots and corresponding trim-and-fill results are presented in the supplementary figures.

## Results

### Literature search process and results

**Fig. 1:**
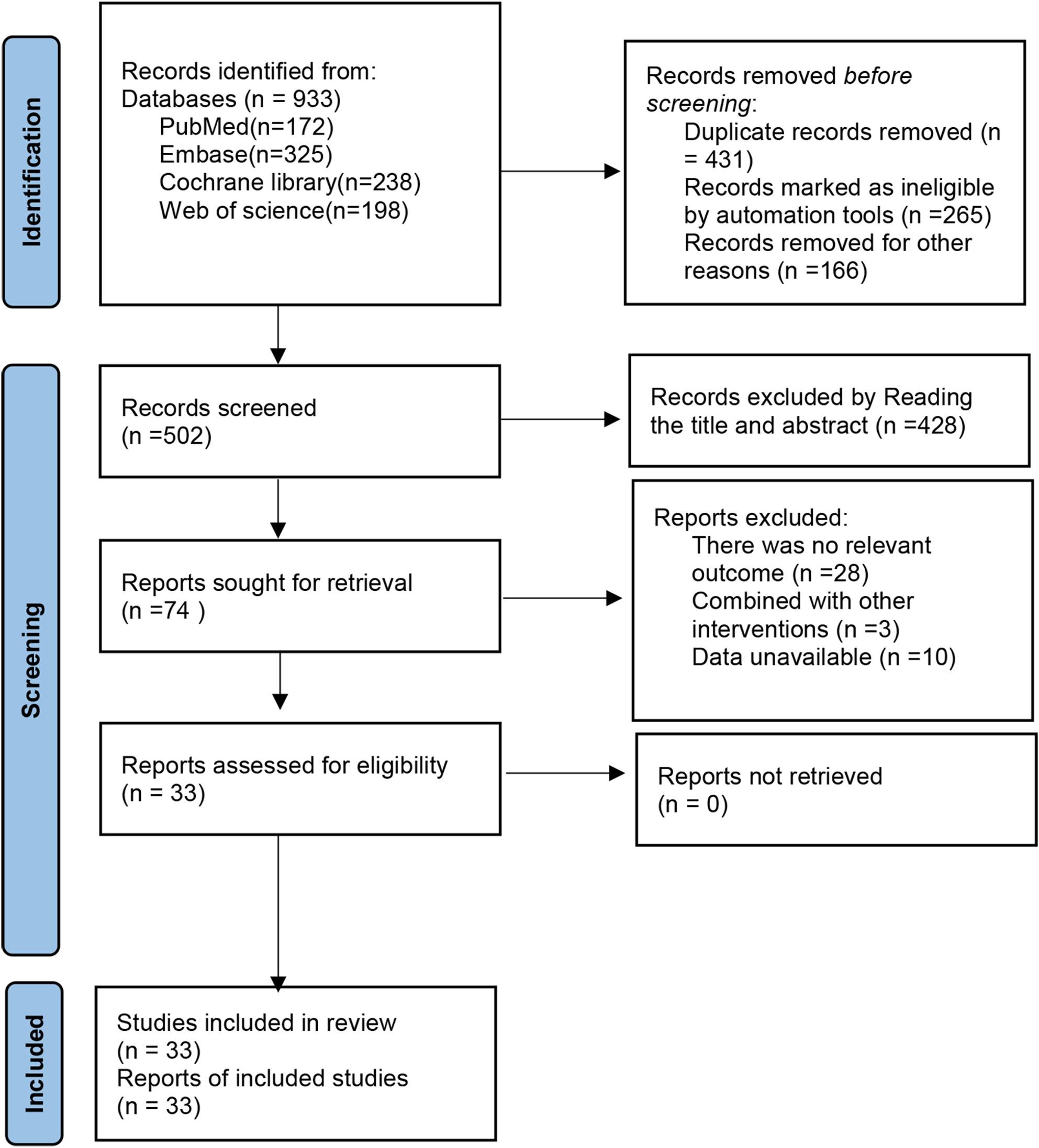
Flow chart of literature screening

The PRISMA flow chart (Fig. 1) shows how studies were selected. A total of 933 relevant articles were retrieved from the databases: 238 from the Cochrane Library, 325 from EMBASE, 198 from Web of Science, and 172 from PubMed. After removing 431 duplicates, 428 articles were excluded based on title and abstract screening. After full-text review of 41 articles, 28 were excluded for lacking relevant outcomes, 3 were excluded because they combined other interventions, and 10 were excluded due to unavailable data. Ultimately, 33 randomized controlled trials [15–47] met the eligibility criteria and were analyzed in this meta-analysis.

### Basic characteristics

The studies selected for inclusion comprised 33 randomized controlled trials published between 2006 and 2025, involving 2,231 participants undergoing surgical procedures. In the experimental groups, stellate ganglion block was used for postoperative pain management, whereas the control groups received placebo treatment. The mean age in the stellate ganglion block groups ranged from 28 to 74.08 years, and from 31 to 75.6 years in the control groups. Three studies involved cardiac surgery, three involved breast cancer surgery, 11 involved orthopedic surgery, five involved thoracic surgery, five involved abdominal surgery, two involved neurosurgery, one involved vascular surgery, and three involved gynecologic surgery. All 33 control groups received placebo treatment (Table 1).

**Table 1:** Basic characteristics

Study	Year	Country	Sample size		ultrasound	Block level	Gender (M/F)	Type of surgery	Mean age		Intervention		Outcomes
			EG	CG	YES/NO	C6/C7/ not specified			EG	CG	EG	CG	
Abd Allah, E	2020	Egypt	20	20	YES	not specified	34/6	Cardiac	53.95	55.25	2% lidocaine 10 ml	No intervention	F1; F3; F4
Salman,A.S	2021	Egypt	40	40	YES	C6	0/80	Breast	51.5	52.4	0.5%bupivacainepreoper5ml	No intervention	F1; F2; F5F6
Choi,E.M	2015	Korea	20	20	YES	C6	21/19	Orthopedic	47.3	49.1	0.375% levobupivacaine 4 ml	No intervention	F1; F2; F5; F6
Eldemrdash,A.M	2025	Egypt	25	25	YES	C7	34/16	Orthopedic	50.92	53.68	0.25%bupivacaine 7.5 ml	No intervention	F2;
GU ,C	2022	China	36	35	YES	not specified	39/32	Thoracic	68.01	67.97	0.5% ropivacaine7 ml	No intervention	F1; F2; F5; F6; F7
Guo,W	2014	China	20	20	NO	not specified	20/20	Thoracic	56	55	1% lidocaine0.12 ml/kg	No intervention	F1
Kim,M.K	2018	Korea	26	26	YES	C6	26/26	Orthopedic	41.15	46.89	0.5% mepivacaine5 ml	No intervention	F1
Kumar,N	2014	India	15	15	YES	C7	22/8	Orthopedic	37.5	38.6	2%lidocaine 3ml	No intervention	F5
Lao,W.L	2024	China	30	28	YES	C6	37/21	Partial hepatectomy	60.23	63.43	0.5% ropivacaine 6ml	No intervention	F1; F2; F3
LI ,X	2022	China	30	30	YES	C6	41/19	Gastrointestinal	59.5	58.4	0.5% ropivacaine7 ml	No intervention	F1
Liu,Y	2025	China	100	100	YES	not specified	0/200	Gynecological	46.16	45.80	0.2% ropivacaine4 ml	No intervention	F1; F2; F5; F7
Liu,D	2025	China	25	25	YES	C6	16/34	Orthopedic	74.08	75.6	0.2-0.375%ropivacaine3-5 ml	No intervention	F1; F2; F3; F5
Luo,D	2023	China	30	30	YES	C6	37/23	Orthopedic	52.87	51.47	0.5% bupivacaine5 ml	No intervention	F1; F2; F5; F7
Morteza,H	2025	Iran	30	30	YES	C6	35/25	**Vascular**	59.1	57.3	5% lidocaine5 ml	No intervention	F2; F5
Park,C.G	2006	Korea	20	20	YES	not specified	25/15	Orthopedic	51.8	52	0.5% **mepivacaine**8ml	No intervention	F5
Peng, ,K	2017	China	20	20	YES	C6	21/19	Orthopedic	44.7	42.5	1% lidocaine6ml	No intervention	F1; F2; F5; F6
Qian,M	2022	China	43	43	YES	C6	39/47	Orthopedic	51.3	53.2	6ml mixture of 0.25% ropivacaine and 1% lidocaine	No intervention	F1; F2;
Rahimzadeh,P	2020	Iran	20	20	YES	not specified	0/40	Gynecological	36.56	32.25	1%lidocaine 10 ml	No intervention	F5
Rajagopalan,V	2020	India	30	30	YES	C6	59/1	Orthopedic	28	31	0.5% bupivacaine10 ml	No intervention	F1; F6
Sermeus,LA	2018	Belgium	10	10	YES	C6	12/8	Orthopedic	54	51	0.5% levobubivacaine5 ml (CPVB),0.5%levobubivacaine3 ml (SGB),	No intervention	F1
Lu,D.H	2024	China.	28	29	YES	C6	37/20	Colorectal cancer s	57.47	56.28	0.2% ropivacaine10 ml	No intervention	F1; F3; F5
Wu,C	2019	China	43	44	YES	not specified	51/36	Thoracic	61.4	59.6	0.5% ropivacaine5 ml	No intervention	F1; F5; F6; F7
Wu,Y	2023	China	30	30	YES	C6	27/33	Neurosurgery	52	51	0.5% ropivacaine (8–10 ml)	No intervention	F2
Wu,Z	2024	China	56	56	YES	C6	58/54	Gastrointestinal	60.04	58.93	0.5% ropivacaine 7 ml	No intervention	F5
Xiang,X	2024	China	37	40	YES	C6	49/28	Thoracic	67.0	68.5	0.35% ropivacaine5ml	No intervention	F1; F2; F3; F5
Yan,S	2023	China	20	20	YES	not specified	23/17	Gastrointestinal	69.5	69.9	0.375% ropivacaine5 ml	No intervention	F1; F5; F7
Yang,R	2023	China	25	23	YES	C6	0/48	Breast	47.72	48.61	0.5% ropivacaine3 ml	No intervention	F1; F2; F3; F5; F7
Yang,X	2022	China	30	30	YES	C6	0/60	Breast	51.4	50.3	0.25% ropivacaine6 ml	No intervention	F1; F5
Yildirim,V	2007	Turkey	50	50	NO	not specified	71/29	Cardiac	52.2	54.5	0.5% ropivacaine10 ml	No intervention	F1; F2; F3; F4
Yuan,X	2025	China	47	47	YES	C6	0/94	Gynecological	51	50	0.2% ropivacaine4 ml	No intervention	F1; F2; F5; F6
Zhang,J	2021	China	50	52	YES	C7	47/55	Neurosurgery	51.43	53.76	0.375%ropivacaine8ml	No intervention	F1
Zhou,W	2025	China	69	69	YES	not specified	93/45	Cardiac	64.1	64.6	0.375% ropivacaine8ml	No intervention	F1; F4
Zhu,G	2020	China	40	39	YES	C7	42/37	Thoracic	56.54	55.91	0.5% ropivacaine7ml	No intervention	F1

F1: Operation timeF2: ComplicationsF3: Hospital stayF4: ICU stayF5: PainF6: Postoperative opioid consumptionsF7: Sleep data of the patients

### Risk of bias assessment

**Fig. 2:**
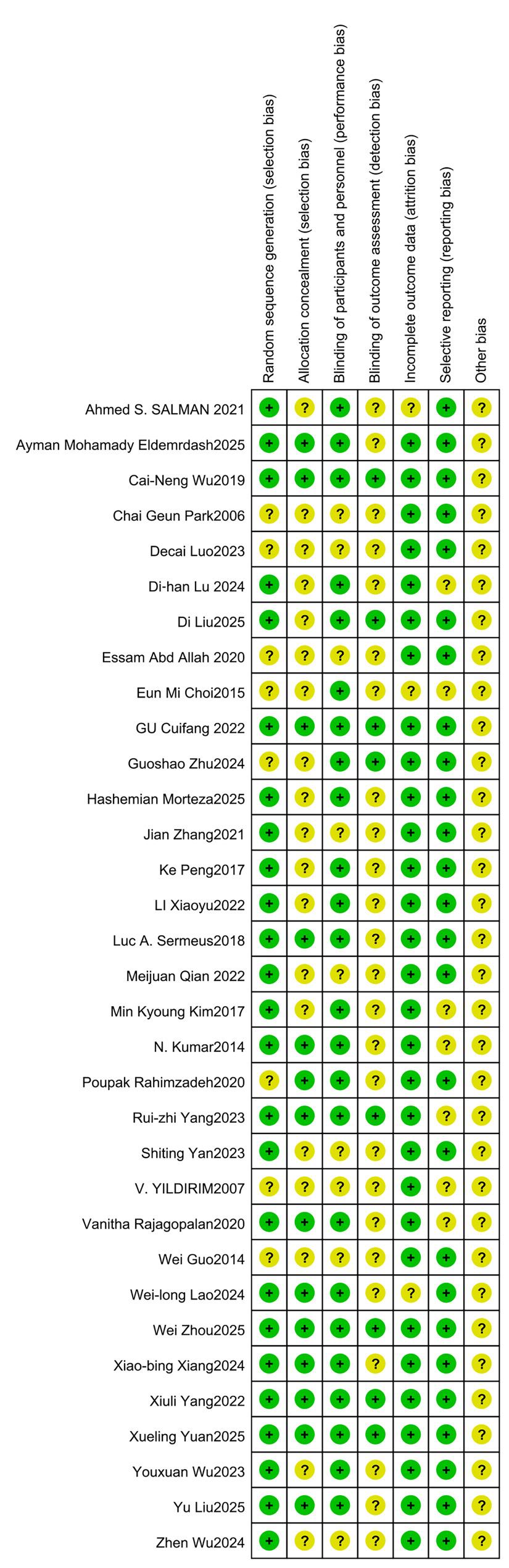
Risk of bias summary

**Fig. 3:**
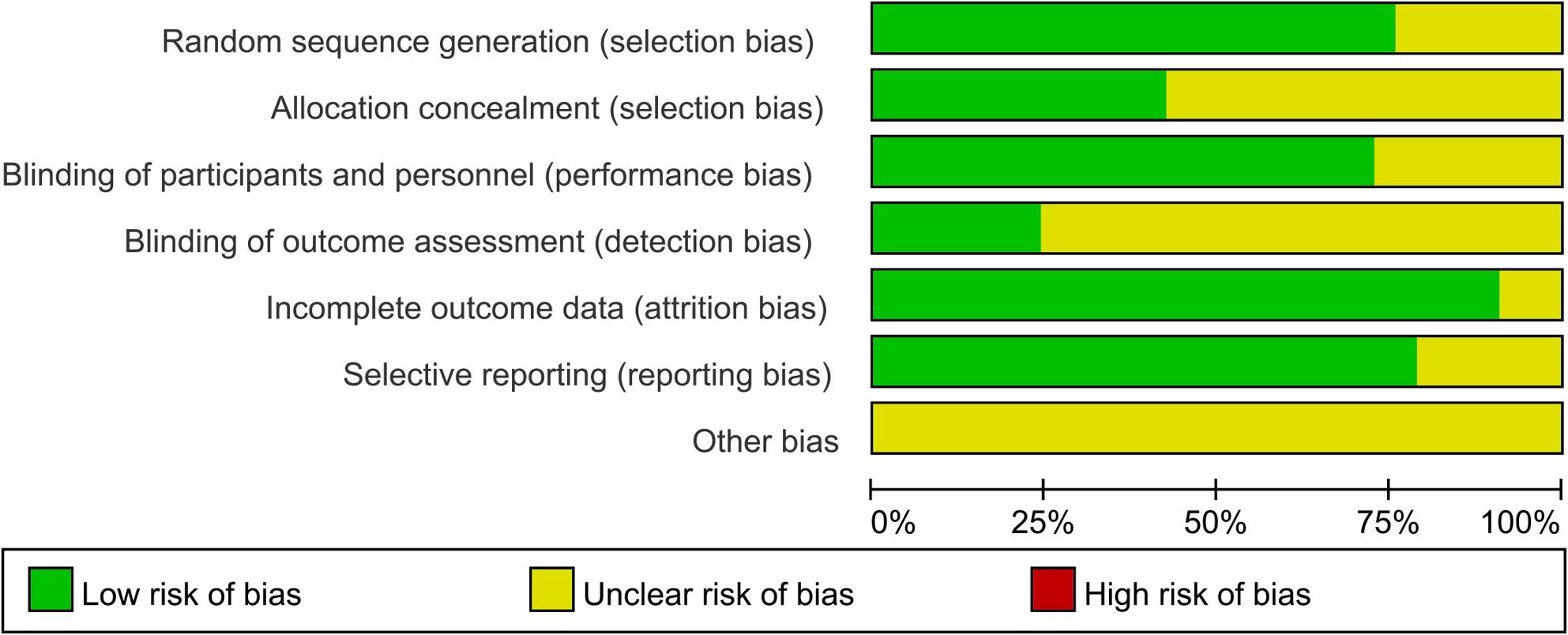
Risk of bias graph

Two reviewers assessed and evaluated multiple sources of bias, such as blinding of outcome assessors, selective reporting, allocation concealment, incomplete outcome data, blinding, random sequence generation, and other biases. The summary of risk of bias and the risk-of-bias graph are presented in Figs. 2 and 3, respectively. As shown, most studies were assessed to have a low risk of bias for random sequence generation, blinding, incomplete outcome data, and selective reporting. Most of the studies were assessed as presenting an unclear risk in terms of allocation concealment and outcome-assessor blinding. Other biases were also rated as unclear risk. None of the studies selected were assessed to have a high risk of bias. The GRADE (Supplementary Table 2) assessment showed high-quality evidence for the effectiveness of stellate ganglion block (SGB) in reducing pain at 6, 12, and 24 h, and for shortening hospital stays. Moderate-quality evidence was found for 48-hour pain, opioid consumption, operative time, ICU stay, and sleep quality, due to heterogeneity and inconsistent results. SGB also significantly reduced postoperative nausea and vomiting but increased the risk of upper eyelid ptosis, both with high-quality evidence. Overall, pain reduction and hospital stay outcomes had the strongest evidence, while other outcomes showed more variability.

### Results of the meta-analysis

#### Primary outcome

##### 6-hour pain score

**Fig. 4:**
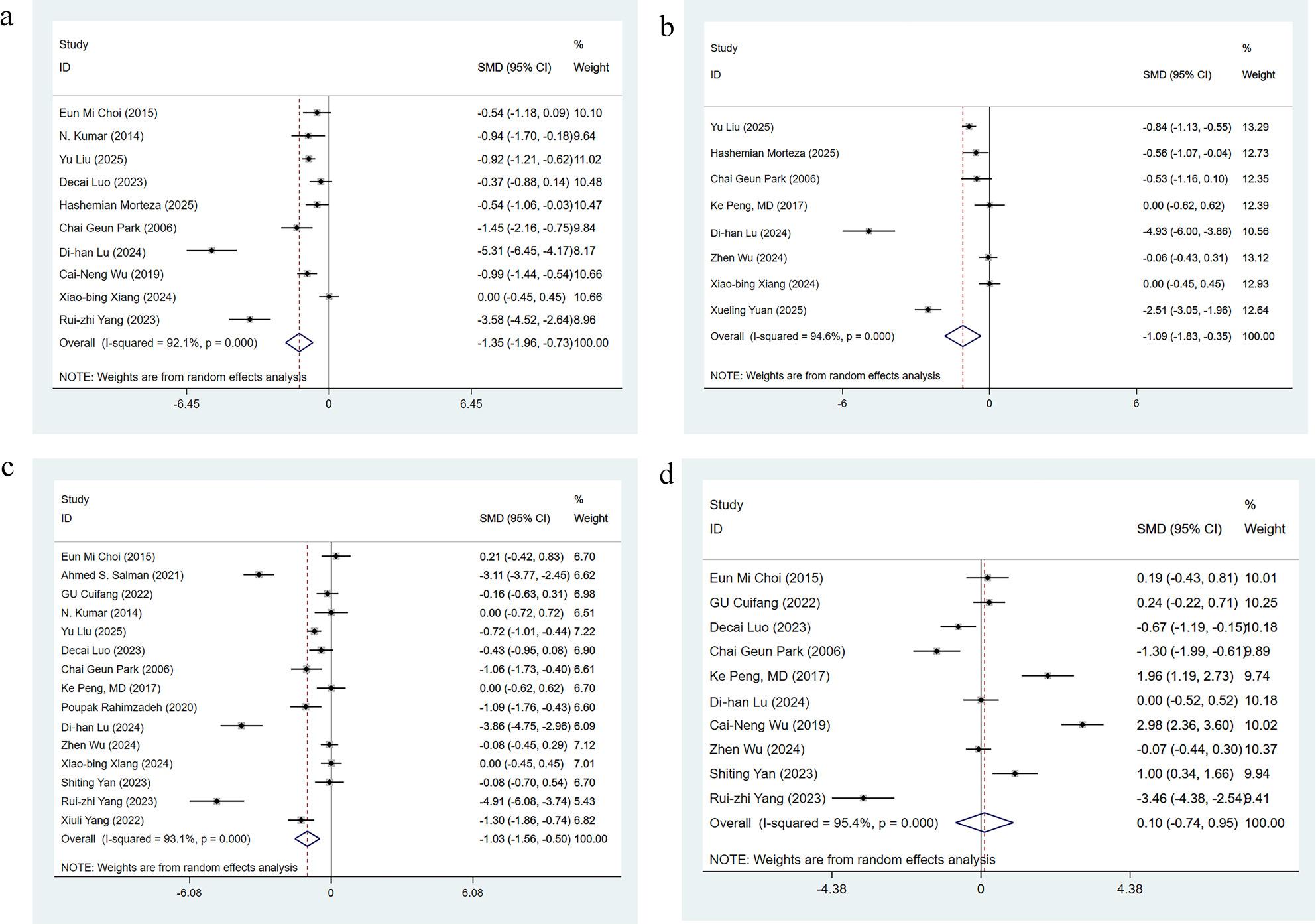
**a** Forest plot of 6h pain scores; (**b**) Forest plot of 12h pain scores; (**c**) Forest plot of 24h pain scores; (**d**) Forest plot of 48h pain scores

Ten randomized controlled trials [17, 22, 25, 27–29, 35, 36, 39, 41] were included, including 699 patients who reported 6-hour pain scores. Heterogeneity testing (I²=92.1%,*P* < 0.001); therefore, a random-effects model was applied. The analysis (Fig. 4a) indicated that stellate ganglion block significantly reduced 6-hour pain scores [SMD = − 1.35, 95% CI (− 1.96, − 0.73)]. Sensitivity analyses indicated that the pooled estimate was robust and not driven by any single study (Supplementary Figure S1).

##### 12-hour pain score

Eight randomized controlled trials [25, 28–30, 35, 38, 39, 44] including 680 patients reported 12-hour pain scores. Heterogeneity testing (I²=94.6%,*P* < 0.001); therefore, a random-effects model was applied. The analysis (Fig. 4b) indicated that stellate ganglion block significantly reduced 12-hour pain scores [SMD = − 1.09, 95% CI (− 1.83, − 0.35)]. Sensitivity analyses suggested that no individual study substantially influenced the pooled estimate (Supplementary Figure S2).

##### 24-hour pain score

Fifteen randomized controlled trials [16, 17, 19, 22, 25, 27, 29, 30, 32, 35, 38–42] including 995 patients reported 24-hour pain scores. Heterogeneity testing (I²=93.1%,*P* < 0.001); therefore, a random-effects model was applied. The analysis (Fig. 4c) indicated that stellate ganglion block significantly reduced 24-hour pain scores [SMD = − 1.03, 95% CI (− 1.56, − 0.50)]. Sensitivity analyses demonstrated consistent results following sequential exclusion of individual studies (Supplementary Figure S3).

##### 48-hour pain score

Ten randomized controlled trials [17, 19, 27, 29, 30, 35, 36, 38, 40, 41] including 595 patients reported 48-hour pain scores. Heterogeneity testing (I²= 95.4%,*P* < 0.001); therefore, a random-effects model was applied. The analysis (Fig. 4d) indicated that stellate ganglion block did not reduce 48-hour pain scores [SMD = 0.10, 95% CI (− 0.74, 0.95)]. Sensitivity analyses yielded similar findings, suggesting that the null result was not attributable to any single study (Supplementary Figure S4).

#### Secondary outcomes

##### Postoperative total opioid consumption

**Fig. 5:**
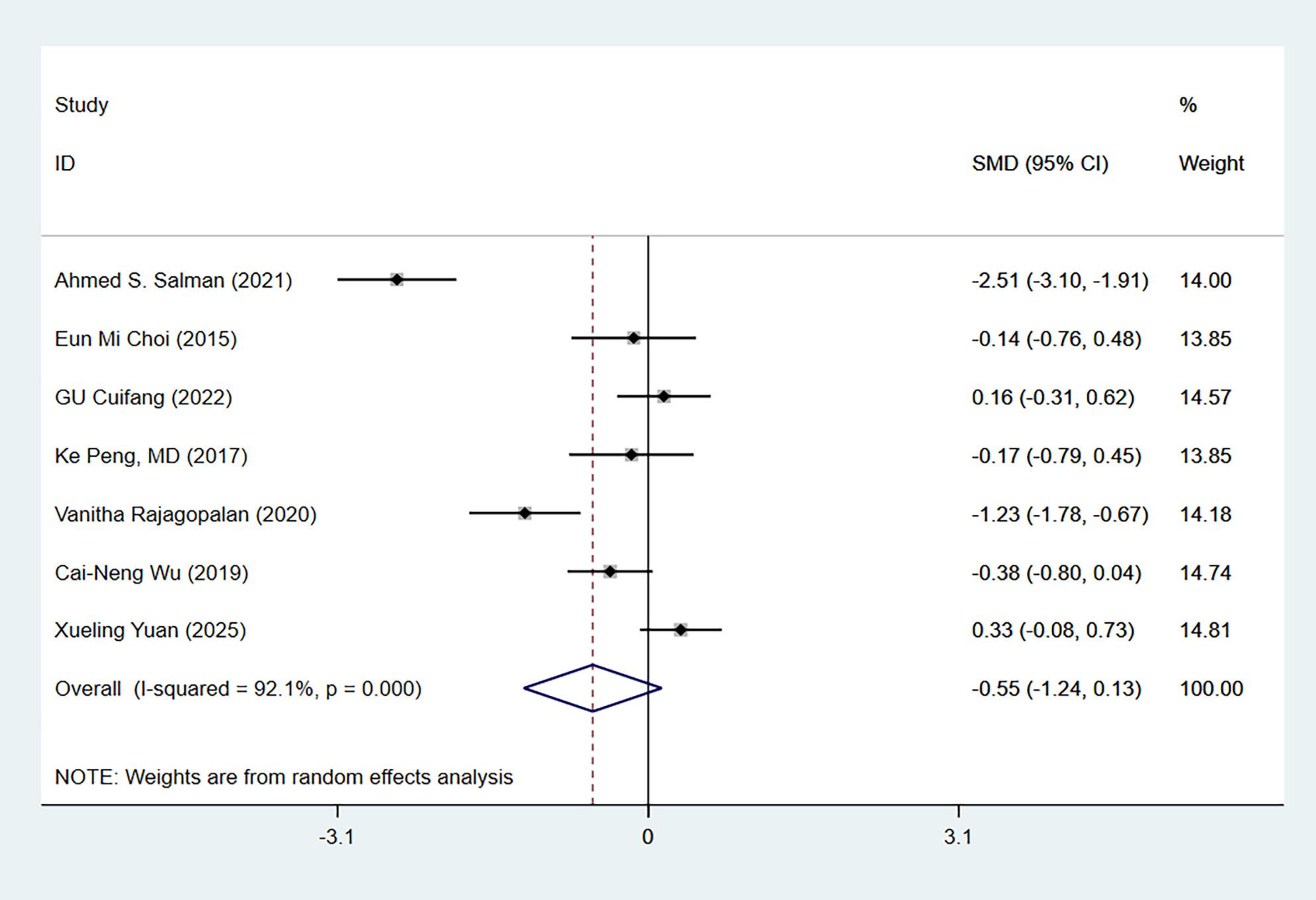
Forest plot of total opioid consumption after surgery

Seven randomized controlled trials [16, 17, 19, 30, 33, 36, 44] including 472 patients reported postoperative total opioid consumption. Heterogeneity testing (I²=92.1%,*P* < 0.001); therefore, a random-effects model was applied. The analysis (Fig. 5) indicated that stellate ganglion block did not affect postoperative total opioid consumption [SMD = − 0.55, 95% CI (− 1.24, 0.13)]. Sensitivity analyses indicated that the results were generally stable and not materially altered by the exclusion of any single study (Supplementary Figure S5).

##### Operative time

**Fig. 6:**
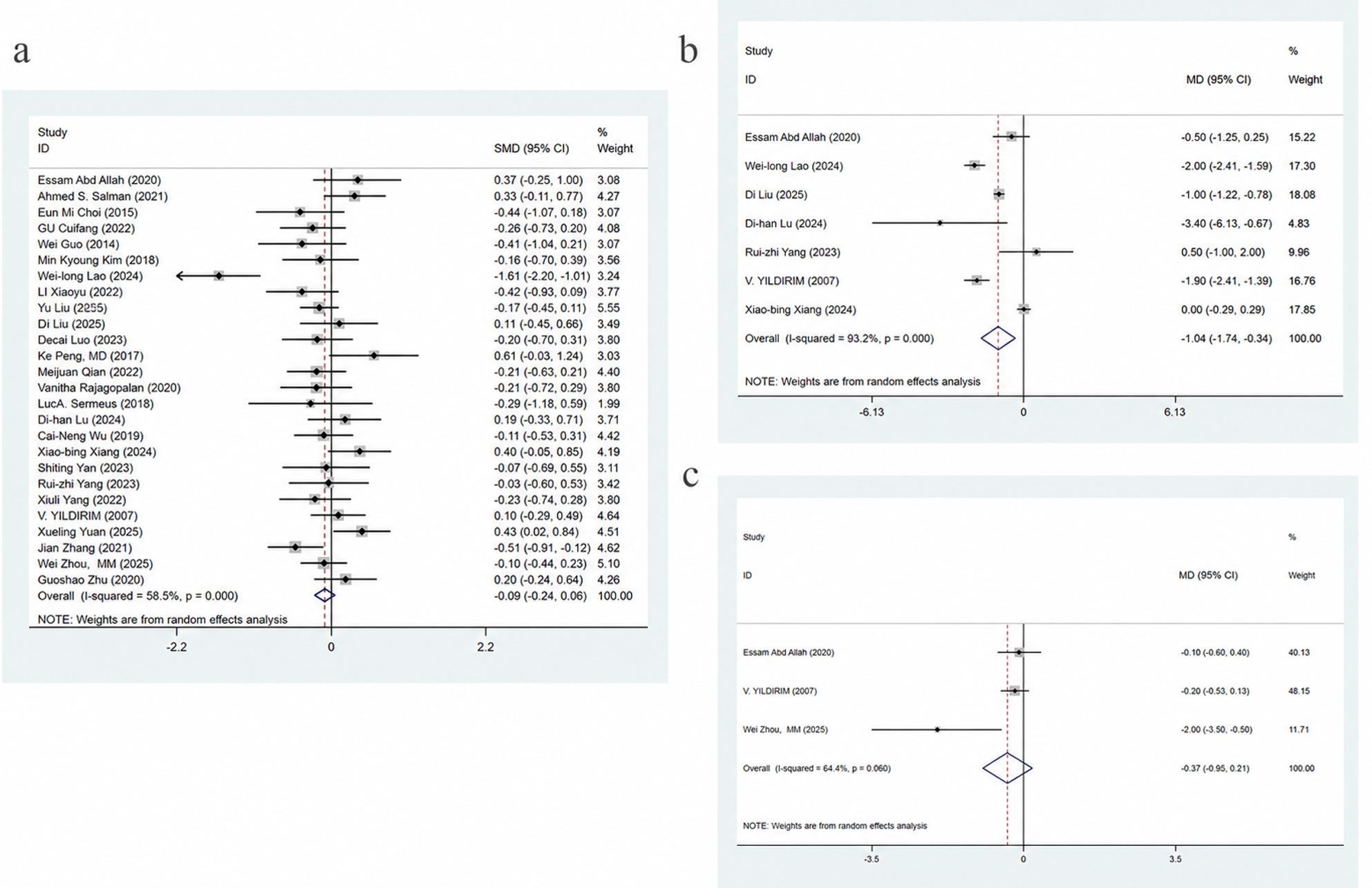
**a**. Forest plot of surgical operation time; (**b**). Forest plot of hospitalization time; (**c**). Forest plot of ICU stay time

Twenty-six randomized controlled trials [15–17, 19–21, 23–27, 30, 31, 33–36, 39–47] including 1,839 patients reported operative time. Heterogeneity testing (I²=58.5%,*P* < 0.001); therefore, a random-effects model was applied. The analysis (Fig. 6a) indicated that stellate ganglion block did not affect operative time [SMD = − 0.09, 95% CI (− 0.24, 0.06)]. Sensitivity analyses indicated that the results were generally stable and not materially altered by the exclusion of any single study (Supplementary Figure S6).

##### Length of hospital stay

Seven randomized controlled trials[15, 23, 26, 35, 39, 41, 43] including 430 patients reported length of hospital stay. Heterogeneity testing (I²=93.2%,*P* < 0.001); therefore, a random-effects model was applied. The analysis (Fig. 6b) indicated that stellate ganglion block significantly *reduced the length of hospital stay* [MD = − 1.04, 95% CI (− 1.74, − 0.34)]. Sensitivity analyses indicated that the results were generally stable and not materially altered by the exclusion of any single study (Supplementary Figure S7).

##### ICU Stay

Three randomized controlled trials [15, 43, 46] including 278 patients reported ICU stay. Heterogeneity testing (I²=64.4%, *P* = 0.001); therefore, a random-effects model was applied. The analysis (Fig. 6c) indicated that stellate ganglion block did not significantly reduce the length of ICU stay [MD = − 0.37, 95% CI (− 0.95, 0.21)]. Sensitivity analyses indicated that the results were generally stable and not materially altered by the exclusion of any single study (Supplementary Figure S8).

##### Sleep quality

**Fig. 7:**
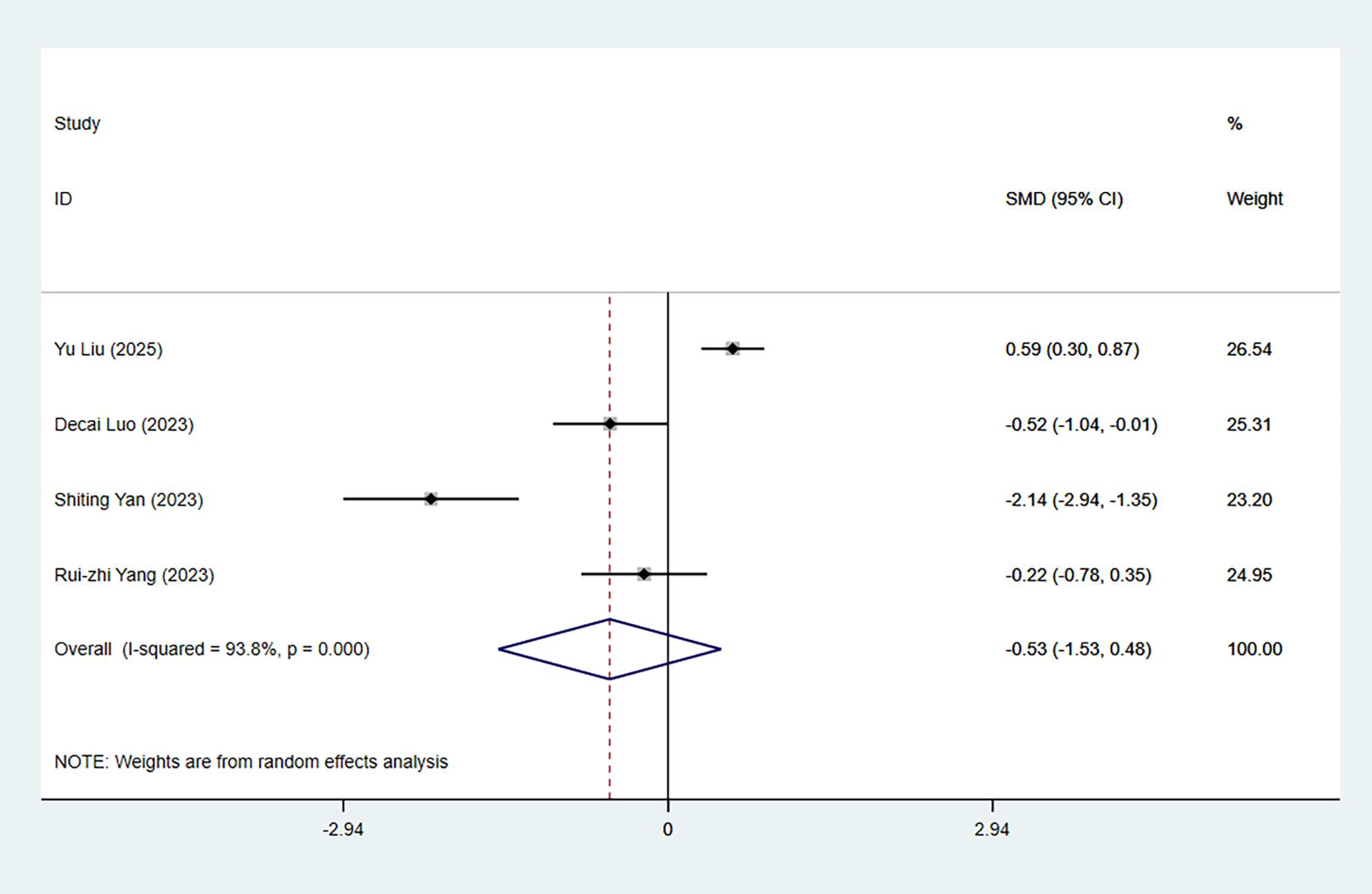
Forest plot of sleep quality

Four randomized controlled trials [25, 27, 40, 41] including 348 patients reported sleep quality. Heterogeneity testing (I² =93.8%,*P* < 0.001); therefore, a random-effects model was applied. The analysis (Fig. 7) indicated that stellate ganglion block did not improve sleep quality [SMD = − 0.53, 95% CI (− 1.53, 0.48)]. Sensitivity analyses indicated that the results were generally stable and not materially altered by the exclusion of any single study (Supplementary Figure S9).

##### Incidence of complications

**Fig. 8:**
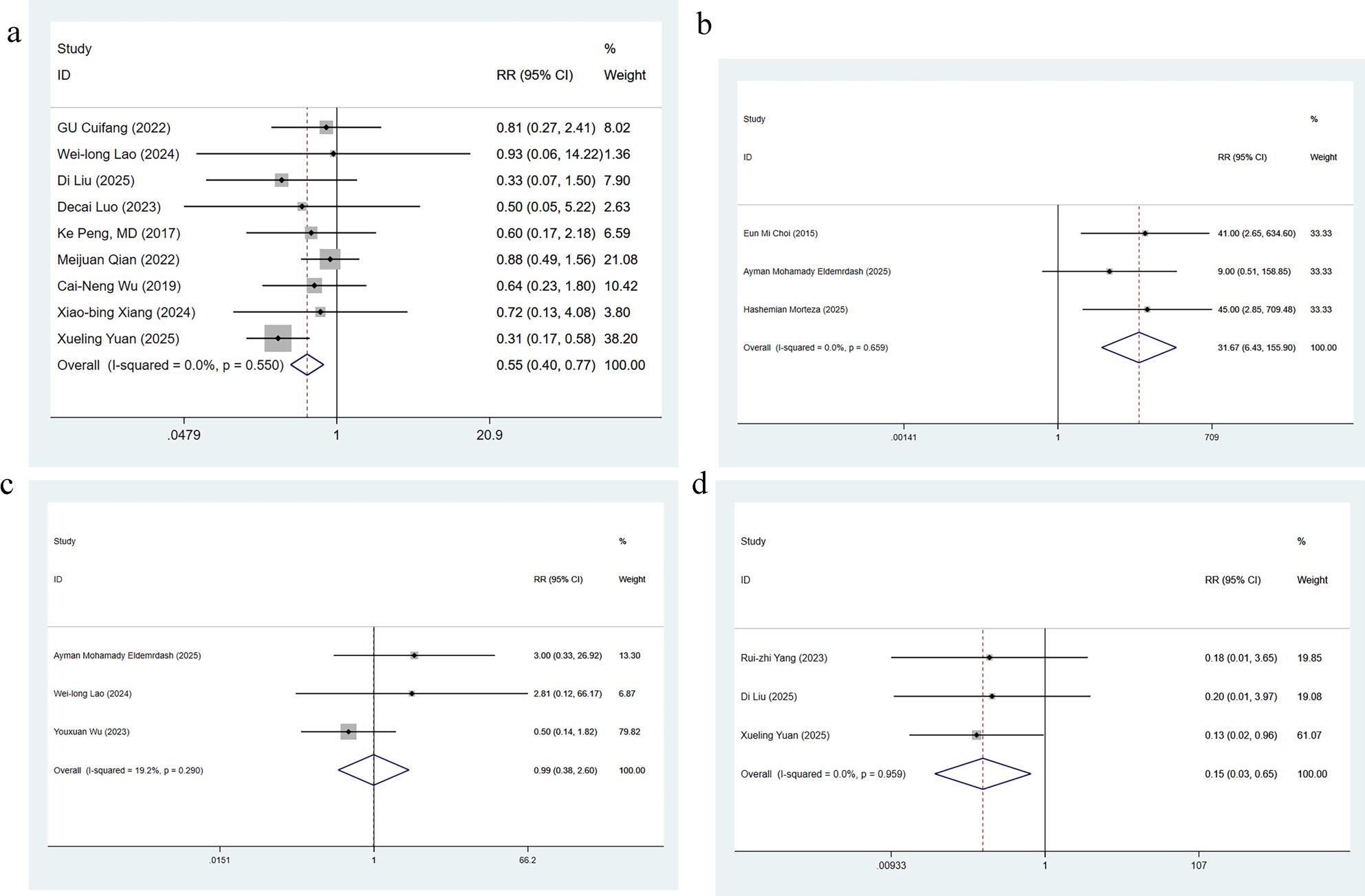
**a**. Forest plot of nausea and vomiting; (**b**). Forest plot of ptosis; (**c**). Forest plot of infect; (**d**). Forest plot of vertigo

Nine randomized controlled trials [19, 23, 26, 27, 30, 31, 36, 39, 44] including 623 patients reported postoperative nausea and vomiting (PONV). Heterogeneity testing (I² = 0.0%, *P* = 0.551); therefore, a fixed-effect model was applied. The analysis (Fig. 8a) indicated that stellate ganglion block significantly reduced the incidence of nausea and vomiting [RR = 0.55, 95% CI (0.40, 0.77)].

Three randomized controlled trials [17, 18, 28] including 150 patients reported upper eyelid ptosis. Heterogeneity testing (I² =0.0%, *P* = 0.659); therefore, a fixed-effect model was applied. The analysis (Fig. 8b) indicated that stellate ganglion block significantly increased the incidence of upper eyelid ptosis [RR = 31.67, 95% CI(6.43, 155.90)].

Three randomized controlled trials [18, 23, 37] including 168 patients reported infection. Heterogeneity testing (I²=19.2%, *P* = 0.200); therefore, a fixed-effect model was applied. The analysis (Fig. 8c) demonstrated that stellate ganglion block did not affect the incidence of infection [RR = 0.99, 95% CI (0.38, 2.60)].

Three randomized controlled trials [26, 41, 44] including 192 patients reported dizziness. Heterogeneity testing (I²=0.0%, *P* = 0.959); therefore, a fixed-effect model was applied. The analysis (Fig. 8d) indicated that stellate ganglion block significantly reduced the incidence of dizziness [RR = 0.15, 95% CI(0.03, 0.65)].

#### Subgroup analysis

Specific subgroup analysis details can be found in Table 2.

**Table 2:** Subgroup analysis results

Outcomes	Group	Subgroup	Number of studies	Heterogeneity (%)	SMD95%CI
6hPain	Surgical type	orthopedics	4	72.7	-0.66(-1.12, -0.2)
		gynaecology	1	0	-1.1(-1.43, -0.77)
		Vascular	1	0	-0.27(-0.52, -0.02)
		breast	1	0	-2(-2.32, -1.68)
		thoracic	2	75.2	-0.06(-0.15, 0.04)
		gastrectomy	1	0	-0.7 (-0.77, -0.63)
	surgical approaches	open	5	94.8	-0.96 (-1.74, -0.18)
		Minimally invasive	5	98.5	-0.42 (-0.76, -0.09)
	ultrasound guidance	YES	9	98	-0.61(-0.91, -0.32)
		NO	1	0	-1.2(-1.7, -0.7)
	block level	C6	6	98	-0.59 (-1.04, -0.15)
		C7	1	0	-1(-1.74, -0.26)
		Not specified	3	96.2	-0.78 (-1.61, 0.05)
	block timing	Before anesthesia	7	87.9	-0.33(-0.52, -0.14)
		Before surgery after anesthesia	3	96.8	-1.29 (-2.2, -0.39)
Outcomes	Group	Subgroup	Number of studies	Heterogeneity (%)	SMD95%CI
12hPain	Surgical type	orthopedics	2	56.5	-0.19 (-0.66, 0.29)
		gynecological	2	0	-1(-1.14, -0.86)
		gastrectomy	2	79.5	-1(-1.14, -0.86)
		thoracic	1	0	0 (-0.11, 0.11)
		Vascular	1	0	-0.24(-0.46, -0.02)
	surgical approaches	open	3	32	-0.19 (-0.41, 0.03)
		Minimally invasive	5	96.8	-0.51 (-0.86, -0.16)
	ultrasound guidance	YES	7	95.7	-0.4(-0.68, -0.12)
		NO	1	0	-0.5 (-1.08, 0.08)
	block level	C6	6	96.1	-0.31 (-0.61, -0.02)
		Not specified	2	54.2	-0.81 (-1.28, -0.33)
	block timing	Before anesthesia	6	96	-0.38 (-0.81, 0.04)
		Before surgery after anesthesia	2	0	-0.5 (-0.55, -0.45)
Outcomes	Group	Subgroup	Number of studies	Heterogeneity (%)	SMD95%CI
24hPain	Surgical type	orthopedics	5	69.7	-0.21(-0.56, 0.14)
		gynecological	2	0	-1.08(-1.42, -0.74)
		gastrectomy	3	94	-0.21(-0.6, 0.17)
		thoracic	2	0	-0.02(-0.12, 0.09)
		breast	3	98.4	-1.5(-2.55, -0.44)
	surgical approaches	open	7	97.7	-0.86(-1.57, -0.16)
		Minimally invasive	8	93.1	-0.3(-0.56, -0.04)
	ultrasound guidance	YES	14	97.1	-0.54 (-0.86, -0.23)
		NO	1	0	-1.2 (-1.89, -0.51)
	block level	C6	9	98	-0.59 (-1.01, -0.17)
		C7	1	0	0 (-0.46, 0.46)
		Not specified	5	90	-0.64 (-1.11, -0.17)
	Block timing	before anesthesia	12	95	-0.42 (-0.74, -0.09)
		before surgery after anesthesia	3	98.8	-1.23 (-2.4, -0.06)
Outcomes	Group	Subgroup	Number of studies	Heterogeneity (%)	SMD95%CI
48hPain	Surgical type	orthopedics	4	94	-0.25 (-1.06, 0.56)
		gastrectomy	3	79.9	=0.07(-0.11, 0.25)
		thoracic	2	0	0.2 (0.17, 0.23)
		breast	1	0	-2 (-2.31, -1.69)
	surgical approaches	open	4	98.6	-0.81 (-2.17, 0.55)
		Minimally invasive	6	92.3	0.12 (-0.01, 0.25)
	ultrasound guidance	YES	9	97.1	-0.11 (-0.3, 0.09)
		NO	1	0	-1.2 (-1.76, -0.64)
	block level	C6	6	97.6	-0.32 (-0.83, 0.19)
		Not specified	4	87.8	0.03 (-0.22, 0.27)
	Block timing	Before anesthesia	7	80.9	0.15 (0, 0.31)
		Before surgery after anesthesia	3	98.8	-1.06(-2.54, 0.42)
Outcomes	Group	Subgroup	Number of studies	Heterogeneity (%)	SMD95%CI
postoperative total opioid consumption	Surgical type	breast surgery	1	0	-5(-5.87,-4.13)
		orthopedics surgery	3	0	-1.5(-2.11,-0.9)
		thoracic surgery	2	72.2%	-1.6(-6.79,3.59)
		gynecological	1	0	1(-0.23,2.23)
	surgical approaches	open	3	95.3	-3.28(-6.67,0.11)
		Minimally invasive	4	36.5	0.36(-1.06,1.79)
	ultrasound guidance	YES	6	93.9	-2(-5.66,1.65)
		NO	1	93	-1.5(-2.11,-0.89)
	block level	C6	5	94.4	-1.95(-4.88,0.98)
		Not specified	2	72.2	-1.6(-6.79,3.59)
Outcomes	Group	Subgroup	Number of studies	Heterogeneity (%)	SMD95%CI
Operation time	Surgical type	cardiac	3	0	0.36(-0.22,0.95)
		breast	3	0	0.19(-0.07,0.46)
		orthopedics	7	0	-1.92(-5.66,1.82)
		thoracic	6	43.2	2.02(-4.96,8.99)
		partial hepatectomy	1	0	-55(-72.53,-37.47)
		gastrectomy	2	0	-8.04(-17.65,1.57)
		gynecological	2	78.8	0.47(-4.9,5.84)
		colorectal	1	0	16.34(-28.32,61)
		neurosurgery	1	0	-13.14(-23.11,-3.17)
	surgical approaches	open	13	25.1	0.2(-0.49,0.88)
		Minimally invasive	13	78.5	-5.19(-11.2,0.81)
	ultrasound guidance	YES	23	68.2	-0.52(-1.98,0.94)
		NO	3	65.3	-4.41(-16.2,7.38）
	block level	C6	16	74.3	-1.96(-5.3,1.39)
		C7	2	76.1	-3.78(-24.6,17.03)
		Not specified	8	0	0.27(-0.31,0.85)
	block timing	before anesthesia	21	68.8	-0.38(-1.88,1.12)
		before surgery after anesthesia	5	43.2	-3.2(-10.08,3.69)
Outcomes	Group	Subgroup	Number of studies	Heterogeneity (%)	WMD95%CI
length of hospital stay	Surgical type	cardiac	2	89.1	-1.23(-2.6,0.14)
		partial hepatectomy	1	0	-2(-2.41-1.59)
		orthopedics	1	0	-1(-1.22,-0.78)
		colorectal	1	0	-3.4(-6.13,-0.67)
		breast	1	0	0.5(-1,2)
		Thoracic	1	0	0(-0.29,0.29)
	surgical approaches	open	4	82.3	-0.94(-1.63,-0.25)
		Minimally invasive	3	96.9	-1.49(-3.25,0.28)
	block level	Not specified	1	0	-0.5(-1.25,0.25)
		C6	6	94.3	-1.13(-1.92,-0.35)
	ultrasound guidance	YES	6	93.1	-0.86(-1.62,-0.1)
		NO	1	0	-1.9(-2.41,-1.39)
	block timing	before anesthesia	4	96.3	-1.1(-2.26,0.06)
		before surgery after anesthesia	3	70.5	-0.94(-2.4,0.51)
Outcomes	Group	Subgroup	Number of studies	Heterogeneity (%)	WMD95%CI
ICU stay time	ultrasound guidance	YES	2	81.9	-0.01(-2.75,0.93)
		NO	1	0	-0.2(-0.53,0.13)
	block timing	before anesthesia	2	0	-0.17(0.45,0.11)
		before surgery after anesthesia	1	0	-2(-3.5,-0.5)
Outcomes	Group	Subgroup	Number of studies	Heterogeneity (%)	SMD95%CI
sleep quality	Surgical type	gynecological	1	0	0.5(0.26,0.74)
		orthopedics	1	0	-1.53(-2.99,-0.07)
		gastrectomy	1	0	-1.45(-1.86,-1.04)
		breast	1	0	-0.88(-3.15,1.3)
	surgical approaches	open	2	0	-1.34(-2.57,-0.11)
		Minimally invasive	2	98.5	-0.47(-2.38,1.44)
	block level	Not specified	2	98.5	-0.47(-2.38,1.44)
		C6	2	0	-1.34(-2.57,-0.11)
	block timing	before anesthesia	3	97.1	-0.76(-2.38,0.83)
		before surgery after anesthesia	1	0	-0.88(-3.15,1.39)

In the analysis of 6-hour postoperative pain, significant results were found for orthopedics (4 studies, I^2^ = 72.7%, MD = -0.66 [-1.12, -0.20]). The open surgery group (5 studies, I^2^ = 94.8%, MD = -0.96 [-1.74, -0.18]) and minimally invasive surgery (5 studies, I^2^ = 98.5%, MD = -0.42 [-0.76, -0.09]) also demonstrated significant effects. Additionally, ultrasound guidance (9 studies, I^2^ = 98%, MD = -0.61 [-0.91, -0.32]) was associated with improved pain outcomes. In terms of block level, the C6 group (6 studies, I^2^ = 98%, MD = -0.59 [-1.04, -0.15]) showed significant pain relief, while block timing indicated that administering blocks before anesthesia (7 studies, I^2^ = 87.9%, MD = -0.33 [-0.52, -0.14]) or before surgery after anesthesia (3 studies, I^2^ = 96.8%, MD = -1.29 [-2.20, -0.39]) contributed to better outcomes.

For 12-hour postoperative pain, the gynecology group (2 studies, MD = -1.00 [-1.14, -0.86]) and gastrectomy (2 studies, I^2^ = 79.5%, MD = -1.00 [-1.14, -0.86]) exhibited significant pain reductions. Minimally invasive surgery (5 studies, I^2^ = 96.8%, MD = -0.51 [-0.86, -0.16]) and ultrasound guidance (7 studies, I^2^ = 95.7%, MD = -0.40 [-0.68, -0.12]) were also associated with improved outcomes. The C6 block level (6 studies, I^2^ = 96.1%, MD = -0.31 [-0.61, -0.02]) and timing of blocks before anesthesia (6 studies, I^2^ = 96%, MD = -0.38 [-0.81, 0.04]) significantly alleviated pain.

For 24-hour postoperative pain, the gynecology group (2 studies, MD = -1.08 [-1.42, -0.74]) and breast surgery group (3 studies, I^2^ = 98.4%, MD = -1.50 [-2.55, -0.44]) showed significant pain reduction. Significant results were also seen for surgical approaches, with open surgery (7 studies, I^2^ = 97.7%, MD = -0.86 [-1.57, -0.16]) and minimally invasive surgery (8 studies, I^2^ = 93.1%, MD = -0.30 [-0.56, -0.04]). Ultrasound guidance (14 studies, I^2^ = 97.1%, MD = -0.54 [-0.86, -0.23]) continued to show benefit. In terms of block level, C6 (9 studies, I^2^ = 98%, MD = -0.59 [-1.01, -0.17]) was the most effective, and block timing showed that blocks administered before anesthesia (12 studies, I^2^ = 95%, MD = -0.42 [-0.74, -0.09]) or before surgery after anesthesia (3 studies, I^2^ = 98.8%, MD = -1.23 [-2.40, -0.06]) were beneficial.

In 48-hour postoperative pain, the open surgery group (4 studies, I^2^ = 98.6%, MD = -0.81 [-2.17, 0.55]) and minimally invasive surgery (6 studies, I^2^ = 92.3%, MD = 0.12 [-0.01, 0.25]) showed varied effects. Ultrasound guidance (9 studies, I^2^ = 97.1%, MD = -0.11 [-0.30, 0.09]) was associated with better outcomes. Block level results for C6 (6 studies, I^2^ = 97.6%, MD = -0.32 [-0.83, 0.19]) and block timing before anesthesia (7 studies, I^2^ = 80.9%, MD = 0.15 [0.00, 0.31]) were significant.

For postoperative total opioid consumption, the orthopedics surgery group (3 studies, MD = -1.50 [-2.11, -0.90]) and open surgery (3 studies, I^2^ = 95.3%, MD = -3.28 [-6.67, 0.11]) showed significant reductions. Ultrasound guidance (6 studies, I^2^ = 93.9%, MD = -2.00 [-5.66, 1.65]) and block level for C6 (5 studies, I^2^ = 94.4%, MD = -1.95 [-4.88, 0.98]) also contributed to opioid consumption reductions.

Regarding operation time, minimally invasive surgery (13 studies, I^2^ = 78.5%, MD = -5.19 [-11.2, 0.81]) showed a significant effect. Ultrasound guidance (23 studies, I^2^ = 68.2%, MD = -0.52 [-1.98, 0.94]) had a more modest impact.

For length of hospital stay, open surgery (4 studies, I^2^ = 82.3%, MD = -0.94 [-1.63, -0.25]) and minimally invasive surgery (3 studies, I^2^ = 96.9%, MD = -1.49 [-3.25, 0.28]) were effective. Ultrasound guidance (6 studies, I^2^ = 93.1%, MD = -0.86 [-1.62, -0.10]) also showed significant benefits.

In ICU stay time, ultrasound guidance (2 studies, I^2^ = 81.9%, MD = -0.01 [-2.75, 0.93]) had no significant effect.

Finally, for sleep quality, open surgery (2 studies, MD = -1.34 [-2.57, -0.11]) and block timing before anesthesia (3 studies, I^2^ = 97.1%, MD = -0.76 [-2.38, 0.83]) significantly impacted sleep outcomes.

### Meta-regression

The meta-regression analysis revealed several significant findings across various postoperative outcomes (Table 3). In the analysis of total postoperative opioid consumption, significant associations were found with surgical type (*p* = 0.01), surgical approach (*p* = 0.004), block level (*p* = 0.005), and ultrasound guidance (*p* = 0.005), all showing a reduction in opioid consumption. However, the timing of block administration and the year of study were not applicable or did not show any significant results. Regarding operation time, none of the variables, including surgical type, approach, block level, ultrasound guidance, block timing, or year, showed statistically significant effects (all p-values > 0.26). In the regression analysis for length of hospital stay, no significant associations were observed with surgical type, approach, or block timing (all p-values > 0.95). Ultrasound guidance showed a non-significant effect (*p* = 0.28). For ICU stay time, while ultrasound guidance (*p* = 0.397) and block timing (*p* = 1) were not significantly associated with ICU duration, year did not have an impact either. Finally, in sleep quality analysis, surgical type, surgical approach, and block level were not significantly associated with postoperative sleep quality, with all p-values > 0.5. Block timing (*p* = 0.68) and year (*p* = 1) also showed no significant effects.

**Table 3:** Meta-regression results

**1. Pain Scores Regression Analysis**		
Outcomes	Coefficient • (Coef)	p-value
6h Surgical Type	-0.025	0.835
12h Surgical Type	0.108	0.355
24h Surgical Type	-0.12	0.22
48h Surgical Type	-0.07	0.234
6h Surgical Approach	0.52	0.21
12h Surgical Approach	-0.29	0.39
24h Surgical Approach	0.53	0.18
48h Surgical Approach	0.91	0.07
6h Block Level	-0.0986	0.685
12h Block Level	-0.2369	0.22
24h Block Level	-0.0379	0.86
48h Block Level	0.096	0.73
6h Ultrasound Guidance	-2.261	0.49
12h Ultrasound Guidance	0.34	0.95
24h Ultrasound Guidance	-1.057	0.7
48h Ultrasound Guidance	-1.056	0.238
6h Block Timing	-2.547	0.46
12h Block Timing	0.13	0.99
24h Block Timing	-1.706	0.56
48h Block Timing	-1.86	0.64
6h Year	-2.46	1
12h Year	-0.022	1
24h Year	-1.41	1
48h Year	-2.915	1
**2. Total Postoperative Opioid Consumption Regression Analysis**		
Outcomes	Coefficient • (Coef)	p-value
Surgical Type	-0.42	0.01
Surgical Approach	-0.46	0.004
Block Level	-0.466	0.005
Ultrasound Guidance	-0.46	0.005
**3. Operation Time Regression Analysis**		
Outcomes	Coefficient • (Coef)	p-value
Surgical Type	-0.447	0.26
Surgical Approach	-0.340	0.4
Block Level	-0.336	0.41
Ultrasound Guidance	-0.3318	0.41
Block Timing	-0.34	0.4
Year	-0.336	0.996
**4. Length of Hospital Stay Regression Analysis**		
Outcomes	Coefficient • (Coef)	p-value
Surgical Type	-0.199	0.95
Surgical Approach	-0.0216	0.99
Block Level	-1.39	0.46
Ultrasound Guidance	-1.42	0.28
Block Timing	-1.13	0.51
Year	-1.31	0.244
**5. ICU Stay Time Regression Analysis**		
Outcomes	Coefficient (Coef)	p-value
Ultrasound Guidance	-3.5	0.397
Block Timing	-3.25	0.23
Year	-3.25	0.443
**6. Sleep Quality Regression Analysis**		
Outcomes	Coefficient • (Coef)	p-value
Surgical Type	-2.29	0.5
Surgical Approach	-0.977	0.68
Block Level	-0.97	0.68
Ultrasound Guidance	Not applicable	-
Block Timing	-0.977	0.68
Year	-0.74	0.27

#### Publication bias

In this study, Egger’s regression test was used to detect publication bias among the included studies. The findings showed no indication of publication bias for 6-hour (Egger = 0.065), 12-hour (Egger = 0.241), 24-hour (Egger = 0.053), and 48-hour pain scores (Egger = 0.905). No publication bias was observed for total opioid consumption (Egger = 0.502) or postoperative total opioid consumption (Egger = 0.221). Similarly, no publication bias was found for operative time (Egger = 0.671), hospital stay (Egger = 0.705), or ICU stay (Egger = 0.318), nausea and vomiting (Egger = 0.889), upper eyelid ptosis (Egger = 0.119), infection (Egger = 0.287), or dizziness (Egger = 0.081). However, publication bias was found for sleep quality (Egger = 0.041). We have drawn a trim-and-fill funnel plot, supporting the judgment of potential publication bias in this outcome. The funnel plots are presented in Supplementary Figures S10–S23.

## Discussion

Our meta-analysis included 33 randomized controlled trials with a total of 2,231 patients and systematically evaluated the efficacy and safety of stellate ganglion block (SGB) in postoperative pain management. The results that SGB may reduce resting pain scores during the early postoperative period (6, 12, and 24 h), suggesting a potential analgesic effect. However, it is important to note that the pooled results exhibited very high statistical heterogeneity (I² > 90%) across these time points, which limits the certainty of this conclusion. But its effect at 48 h postoperatively was limited. Compared with the control group, there was no statistically significant difference in postoperative total opioid consumption. SGB reduced hospital stay and ICU stay but did not affect operative time. No significant difference in sleep quality was found between the two groups. Regarding safety, SGB markedly decreased the occurrence of nausea and vomiting and dizziness but increased the risk of upper eyelid ptosis. Between the two groups, no significant difference was detected in the incidence of infection.

In this study, stellate ganglion block was found to reduce pain in the early postoperative period (6, 12, and 24 h); This finding aligns with the physiological mechanism whereby SGB achieves widespread autonomic blockade by blocking the cervical sympathetic ganglia with local anesthetics, thereby inhibiting sympathetic nerve transmission in the upper limbs, head and neck, and cardiac regions [48]. however, compared with the control group, there were no statistically significant differences in resting pain scores at 48 h postoperatively. This may be because the duration of action of local anesthetics is insufficient to cover the pain phase beyond 48 h, during which pain becomes predominantly mediated by central sensitization. At that stage, SGB cannot block the activation of NMDA receptors in the dorsal horn of the spinal cord, and other interventions are needed [49]. Previous studies have also confirmed that local anesthetics of different volumes produce varying degrees of sympathetic blockade, and the effects are not permanent [50]. This time limitation suggests that SGB primarily covers the initial phase of acute postoperative pain when it is most intense. Its clinical role should be as an effective component within multimodal analgesia protocols for controlling early breakthrough pain, rather than providing sustained long-term analgesia lasting several days. Although early postoperative pain was reduced, there were no statistically significant differences between the two groups in postoperative total opioid consumption. This indicates that early pain relief alone did not consistently translate into clinically meaningful opioid savings. This seemingly contradictory result may stem from multiple factors. First, the threshold for moderate-to-severe postoperative pain varies significantly among individuals; early effective analgesia may not alter the subsequent incidence of moderate-to-severe pain or the intensity of analgesic demand. Second, variations in rescue analgesia protocols across studies (e.g., opioid type, timing of administration) may have offset the initial savings effect of SGB. Furthermore, patient pain perception and opioid responsiveness exhibit high inherent heterogeneity, reflected in the extremely high heterogeneity (I² > 90%) observed in pain score outcomes in this study. Therefore, the analgesic benefit of SGB may be more evident in enhancing early patient comfort rather than directly reducing total opioid consumption. Regarding recovery indicators, SGB shortened both hospital stay and ICU duration. Notably, SGB had no effect on operative time. This can be related to the nature of the surgeries themselves. In terms of surgical distribution, orthopedic procedures were most common, followed by cardiothoracic and abdominal surgeries, with the remaining procedures being gynecologic, vascular, neurosurgical, and breast surgeries. The wide range of surgical types and varying degrees of procedural difficulty likely contributed to this finding. SGB is usually performed before surgery begins and therefore does not affect operative time. This excludes the possibility that it shortens hospital stays by improving surgical efficiency. Therefore, the reduction in hospital length of stay is more likely attributable to SGB improving the quality of early postoperative recovery. Better early pain control may prompt patients to ambulate earlier, resume oral intake sooner, and reduce the risk of pain-related complications and organ dysfunction, further shortening ICU stays. These represent core elements of the Enhanced Recovery After Surgery (ERAS) concept. This finding highlights the potential value of integrating regional analgesia techniques like nerve blocks into ERAS pathways. By optimizing pain management, this approach accelerates overall recovery and conserves healthcare resources. 

Regarding safety outcomes, SGB reduced the risk of postoperative nausea and vomiting (PONV) and dizziness, with relative risk ratios of 0.55 and 0.15, respectively. PONV is a common postoperative complication associated with multiple factors, including opioid use [51]. This benefit of SGB may relate to its trend toward reduced opioid requirements (though not statistically significant) and the hemodynamic stabilizing effects of sympathetic inhibition. However, SGB also significantly increased the risk of ptosis, a typical and expected complication resulting from the procedure’s impact on the cervical sympathetic trunk, leading to transient Horner’s syndrome [52]. Despite the elevated risk, this complication is typically reversible. Furthermore, analyses indicate that SGB does not increase the risk of postoperative infection, providing preliminary reassurance regarding clinical concerns about potential infection introduction from this invasive procedure. These safety data offer crucial evidence for clinicians weighing the benefits and risks of SGB. In this meta-analysis, only these four complications were reported. This is because other complications were scattered across studies, with sample sizes too small—only one or two studies each—thus lacking clinical relevance. Prior studies have reported that SGB can improve sleep quality [53].However, this meta-analysis showed no statistically significant difference between the two groups. Moreover, this outcome carries potential publication bias. Further analysis of the trimmed funnel plot indicates that the existing evidence is weak. Future studies with rigorous designs and patient-reported outcomes (such as sleep quality) as primary endpoints are needed to explore the impact of SGB on this important quality-of-life indicator [54].

Pain scores at various postoperative time points exhibited extremely high heterogeneity (I² > 90%). To further explore sources of heterogeneity, this meta-analysis conducted subgroup analyses and meta-regression analyses based on surgical type, surgical approach, use of ultrasound guidance, block plane, and timing of block administration. Additionally, meta-regression analysis was performed on publication year. Results indicated that surgical approach, type of surgery (thoracic vs. gastrointestinal), and block plane were sources of heterogeneity in 48-hour postoperative pain scores. The timing of block administration was identified as a potential effect modifier associated with analgesic efficacy at 6 and 48 h postoperatively. This finding points to an important direction for future research: optimizing the timing of SGB implementation (e.g., comparing preoperative prophylactic block with postoperative therapeutic block) may further maximize analgesic benefits and potentially prolong duration of action. Future studies should also conduct stratified analyses based on different surgical types, approaches, and blockade planes to optimize individualized SGB application. Overall, the included studies exhibited low risk of bias, and no significant publication bias was detected for primary outcomes except sleep quality, enhancing the internal validity and credibility of core findings such as early analgesia, shorter hospital stays, and changes in specific complications.

### Limitations

This study has several limitations. First, variations in analgesic regimens among control groups represent a key potential confounder and source of heterogeneity. After careful assessment, we determined that meaningful quantitative subgroup analysis or meta-regression for the complex variable “analgesic regimen” was unfeasible due to highly heterogeneous and incomplete information in the original reports (e.g., most studies described only “standardized analgesia” or “routine protocols”). Forced analysis could yield misleading results. Given the three-dimensional variable of “type, concentration, and volume” of local anesthetics, conducting subgroup analyses with the current limited number of studies (*N* = 33) would result in small sample sizes for each subgroup. Any statistical comparisons would be unreliable due to insufficient power. Therefore, we did not perform quantitative analyses. Currently, there is no universally accepted “optimal dose” hypothesis to guide the analysis and interpretation of differences between subgroups.

Second, despite comprehensive literature searches and risk of bias assessments, most included studies carried an “unclear” risk regarding allocation concealment and outcome assessor blinding. Inadequate allocation concealment may compromise intergroup comparability, while unblinded outcome assessors—particularly when evaluating subjective outcomes like pain scores or postoperative nausea and vomiting—may introduce measurement bias. This could skew estimates of ganglion block analgesic efficacy and complication rates. Evidence indicates that unblinded outcome assessors tend to overestimate the effects of experimental interventions, inflating odds ratios by an average of 29% [55]. This necessitates caution in interpreting these results, as the true effect may be either over- or underestimated.

Third, regarding postoperative sleep quality as an outcome, publication bias implies that some small studies showing negative results may remain unpublished, potentially leading us to overestimate the effects observed in published studies. This strongly suggests that the existing evidence on the impact of stellate ganglion block on postoperative sleep quality is both limited and of low quality.

Finally, the outcomes examined in this study are predominantly short-term, lacking assessment data on long-term patient outcomes such as the incidence of chronic postoperative pain, long-term functional recovery, and quality of life. This limitation prevents us from evaluating whether stellate ganglion block offers long-term benefits beyond acute perioperative pain management [22]. Therefore, current conclusions are restricted to the short-term perioperative effects of stellate ganglion block, with its long-term value and cost-effectiveness requiring further investigation.

## Conclusions

The perioperative application of SGB serves as an effective and relatively safe adjunctive analgesic strategy, capable of alleviating early postoperative acute pain in patients. This provides clinicians with a valuable option when formulating individualized, multimodal analgesic strategies. However, substantial heterogeneity and variability in study designs limit the certainty of these findings. Further high-quality, procedure-specific trials are required. Future research should focus on optimizing SGB technical specifications, evaluating its long-term benefits and risks, and exploring its potential role in preventing pain chronicity to further solidify its position in perioperative management.

## Supplementary Information

Supplementary Material 1.

## Data Availability

The data used to support the findings of this study are included within the article.
