# Supplementary material for: Efficacy and safety of stellate ganglion block for postoperative pain management: a systematic review and meta-analysis of randomized controlled trials
Source: BMC Anesthesiol. 2026 May 11;26:393. doi: 10.1186/s12871-026-03901-0 (PMC13330201; doi:10.1186/s12871-026-03901-0)
Supplement: Supplementary file 1 — Supplementary Material 1. [file 12871_2026_3901_MOESM1_ESM.docx]

**Supplementary materials**

Table S1 Literature search strategy

**1.Pubmed**

| Search number | Query |
| --- | --- |
| #1 | ,(SGB[Title/Abstract]) OR (Stellate ganglion block[Title/Abstract]),,,"""SGB""[Title/Abstract] OR ""stellate ganglion block""[Title/Abstract]","1,955",17:23:33,2025/08/05 |
| #2 | General Surgery[MeSH Terms],,,"""general surgery""[MeSH Terms]","41,557",17:24:51,2025/08/05 |
| #3 | "((General Surgery[Title/Abstract]) OR (Surgery, General[Title/Abstract])) OR (Surgery[Title/Abstract])",,,"""general surgery""[Title/Abstract] OR ""surgery general""[Title/Abstract] OR ""Surgery""[Title/Abstract]","1,669,496",17:26:47,2025/08/05 |
| #4 | "(General Surgery[MeSH Terms]) OR (((General Surgery[Title/Abstract]) OR (Surgery, General[Title/Abstract])) OR (Surgery[Title/Abstract]))",,,"""general surgery""[MeSH Terms] OR ""general surgery""[Title/Abstract] OR ""surgery general""[Title/Abstract] OR ""Surgery""[Title/Abstract]","1,692,229",17:30:33,2025/08/05 |
| #5 | "((SGB[Title/Abstract]) OR (Stellate ganglion block[Title/Abstract])) AND ((General Surgery[MeSH Terms]) OR (((General Surgery[Title/Abstract]) OR (Surgery, General[Title/Abstract])) OR (Surgery[Title/Abstract])))",,,"(""SGB""[Title/Abstract] OR ""stellate ganglion block""[Title/Abstract]) AND (""general surgery""[MeSH Terms] OR (""general surgery""[Title/Abstract] OR ""surgery general""[Title/Abstract] OR ""Surgery""[Title/Abstract]))",172,17:31:39,2025/08/05 |

**2.Cochrane**

| Search number | Query |
| --- | --- |
| #1 | (SGB):ti,ab,kw OR (Stellate ganglion block):ti,ab,kw 648 |
| #2 | MeSH descriptor: [General Surgery] explode all trees 514 |
| #3 | (General Surgery):ti,ab,kw OR (Surgery, General):ti,ab,kw OR (Surgery):ti,ab,kw 309244 |
| #4 | #2or#3 309244 |
| #5 | #1and#4 240 |

**3.Embase**

| Search number | Query |
| --- | --- |
| #1 | ,"'general surgery'/exp",26078,6 Aug 2025 |
| #2 | "'general surgery':ab,ti",26065,6 Aug 2025 |
| #3 | "'surgery, general':ab,ti",940,6 Aug 2025 |
| #4 | "'surgery':ab,ti",2295844,6 Aug 2025 |
| #5 | "#1 OR #2 OR #3 OR #4",2303095,6 Aug 2025 |
| #6 | "'sgb':ab,ti",1806,6 Aug 2025 |
| #7 | "'stellate ganglion block':ab,ti",1724,6 Aug 2025 |
| #8 | "#6 OR #7",2922,6 Aug 2025 |
| #9, | "#5 AND #8",325,6 Aug 2025 |

**4.Web of science**

| Search number | Query |
| --- | --- |
| #1 | TS=(SGB) OR TS=(Stellate ganglion block) 2830 Wed Aug 06 2025 06:07:07 GMT+0800 |
| #2 | TS=(General Surgery)OR TS=(Surgery, General)OR TS=(Surgery) 1690833 Wed Aug 06 2025 06:09:13 GMT+0800 |
| #3 | #2 AND #1 198 Wed Aug 06 2025 06:09:37 GMT+0800 |

Table s2 GARDE outcomes


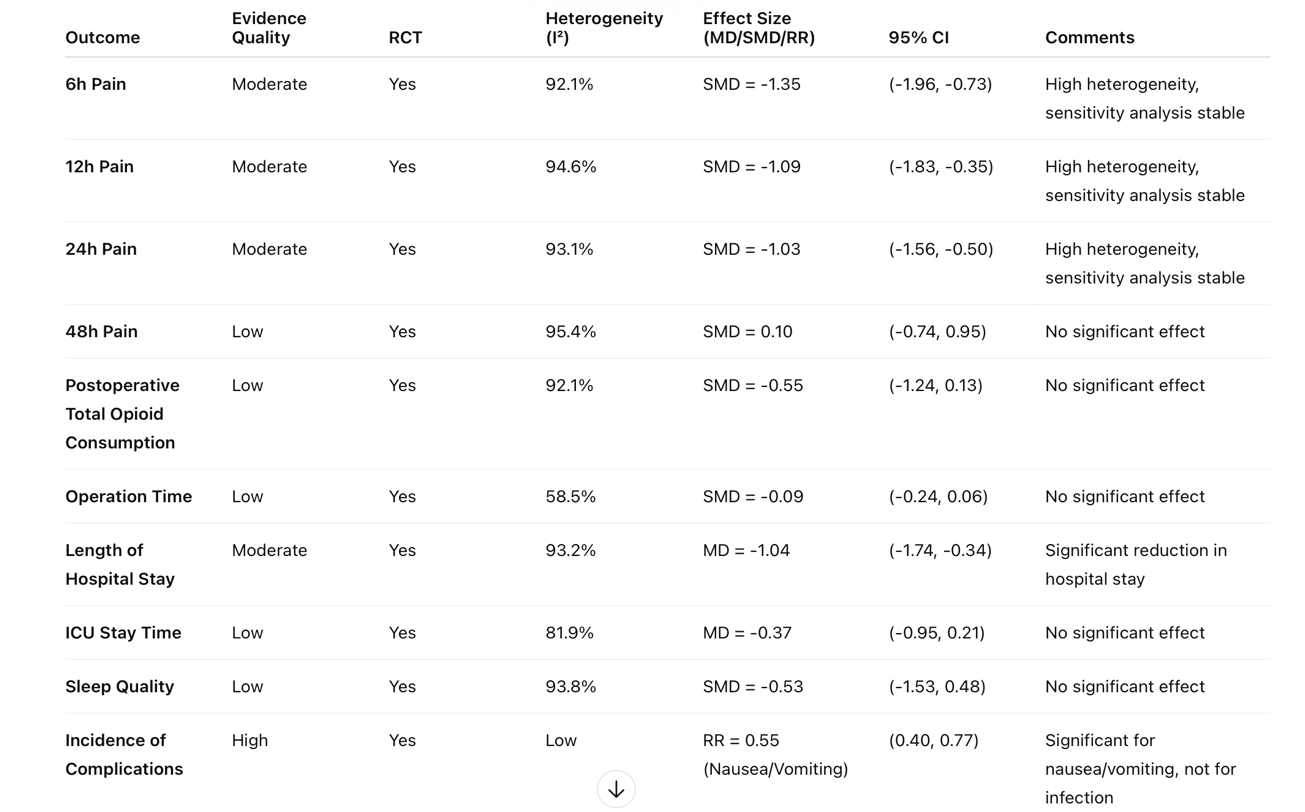


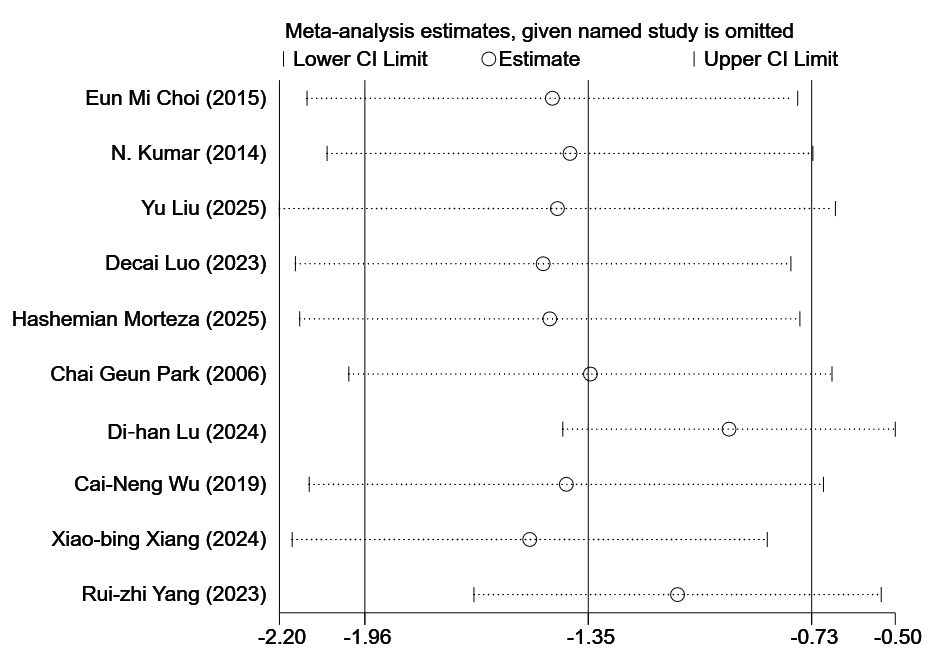


Fig.S1 Sensitivity analysis of 6-hour pain score


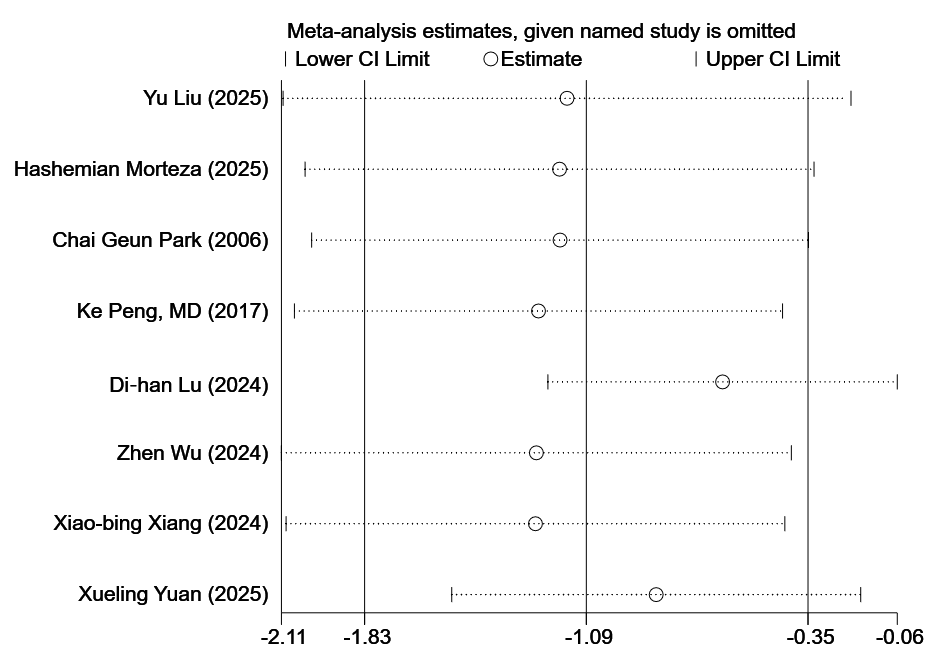


Fig.S2 Sensitivity analysis of 12h pain score


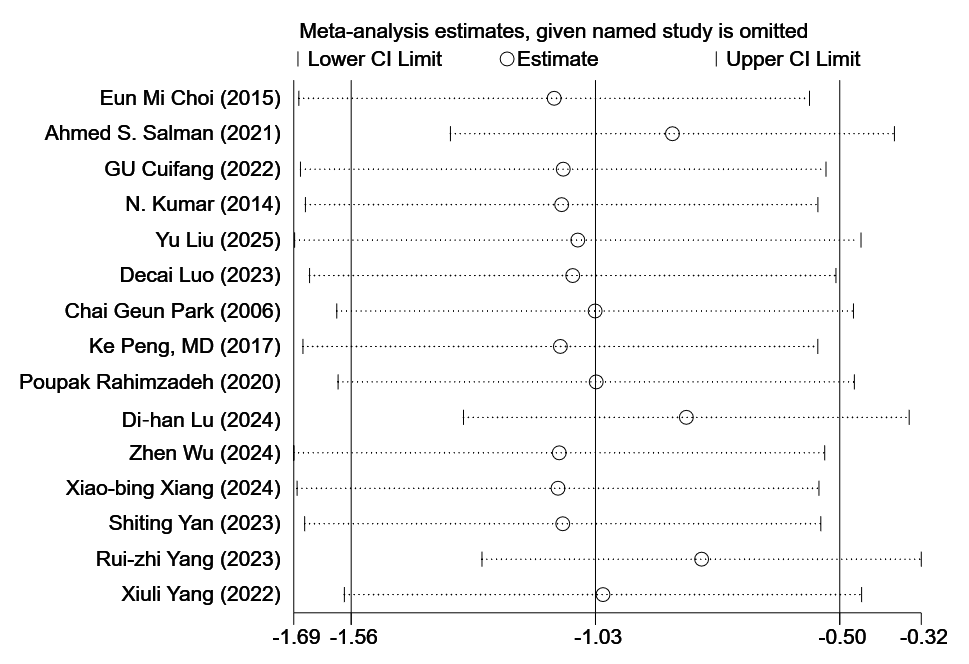


Fig.S3 Sensitivity analysis of 24-hour pain score


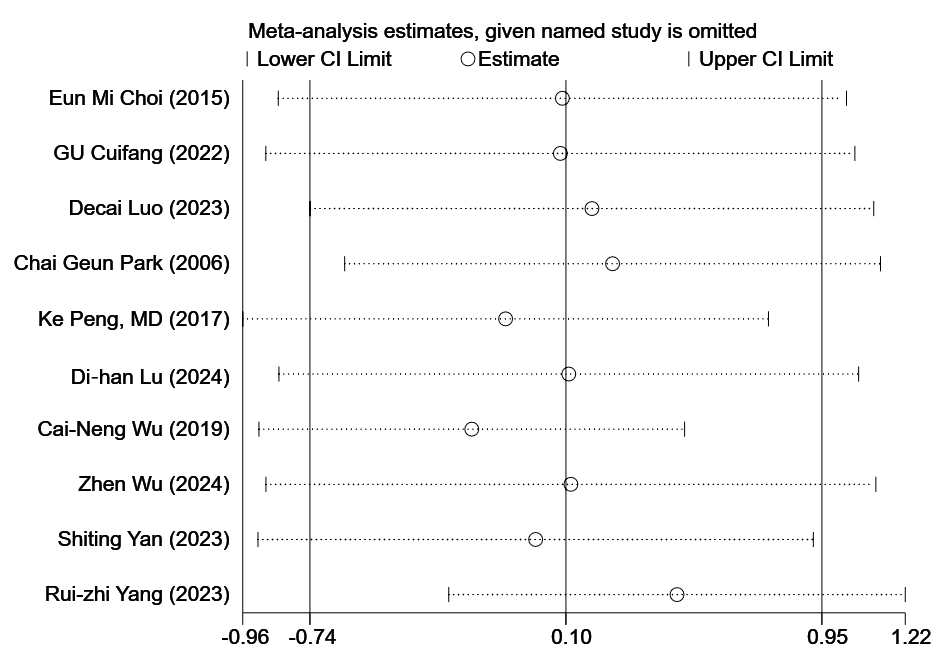


Fig.S4 Sensitivity analysis of 48 hour pain score


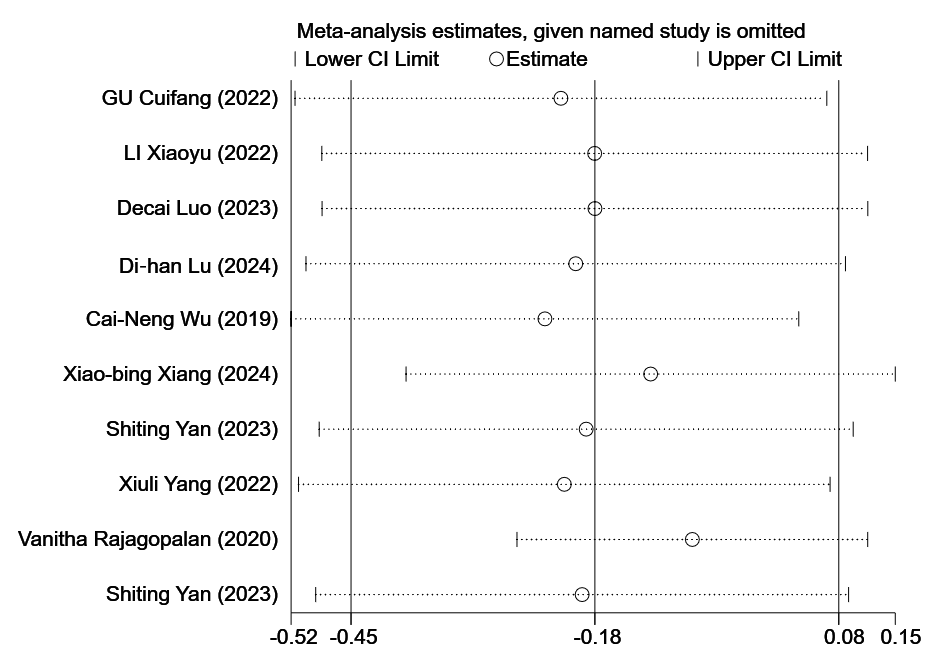


Fig.S5 Sensitivity analysis of total opioid consumption


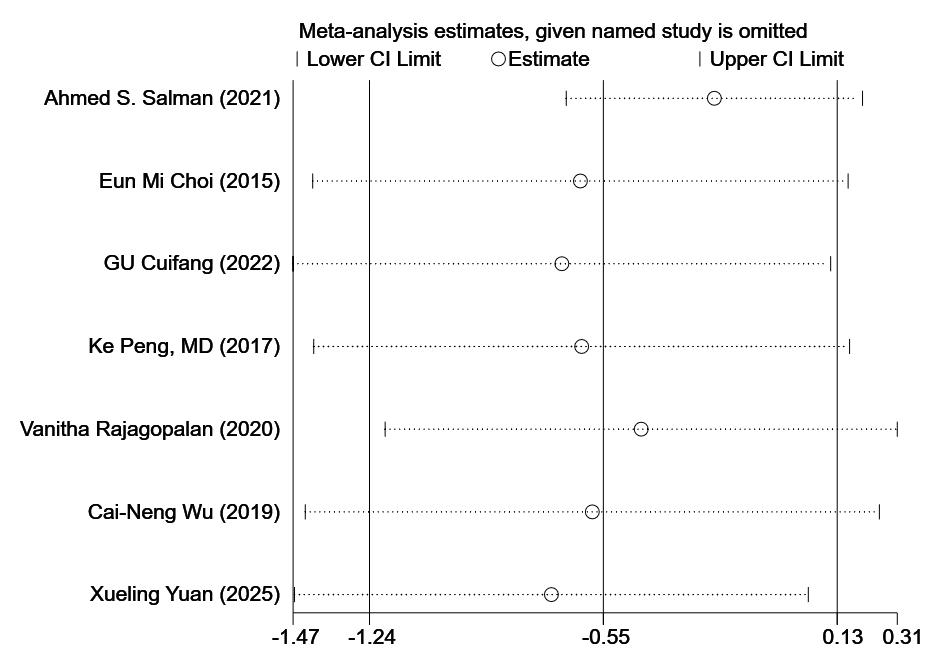


Fig.S6 Sensitivity analysis of total opioid consumption after surgery


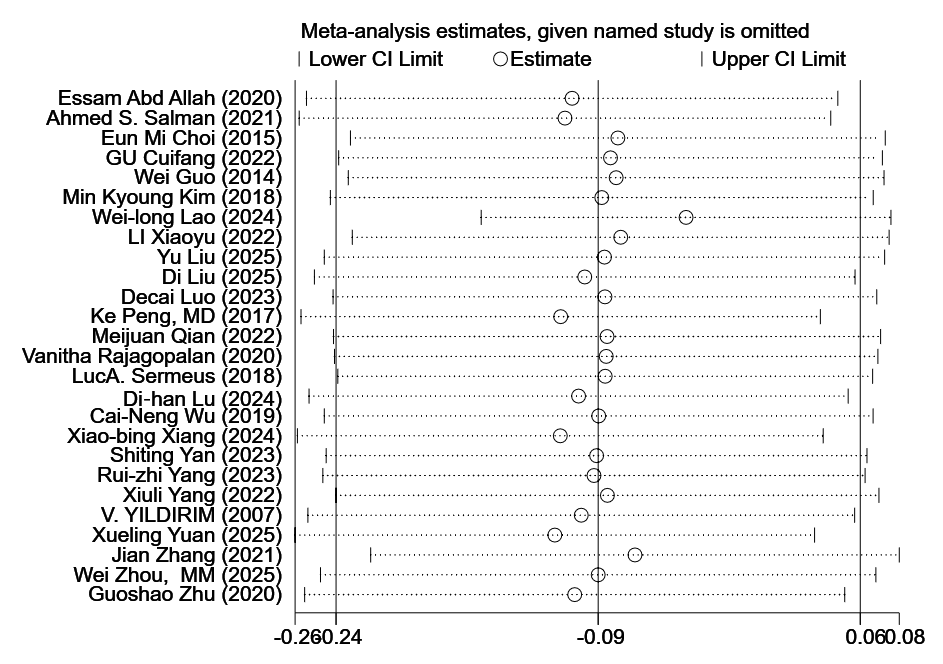


Fig.S7 Sensitivity analysis of surgical operation time


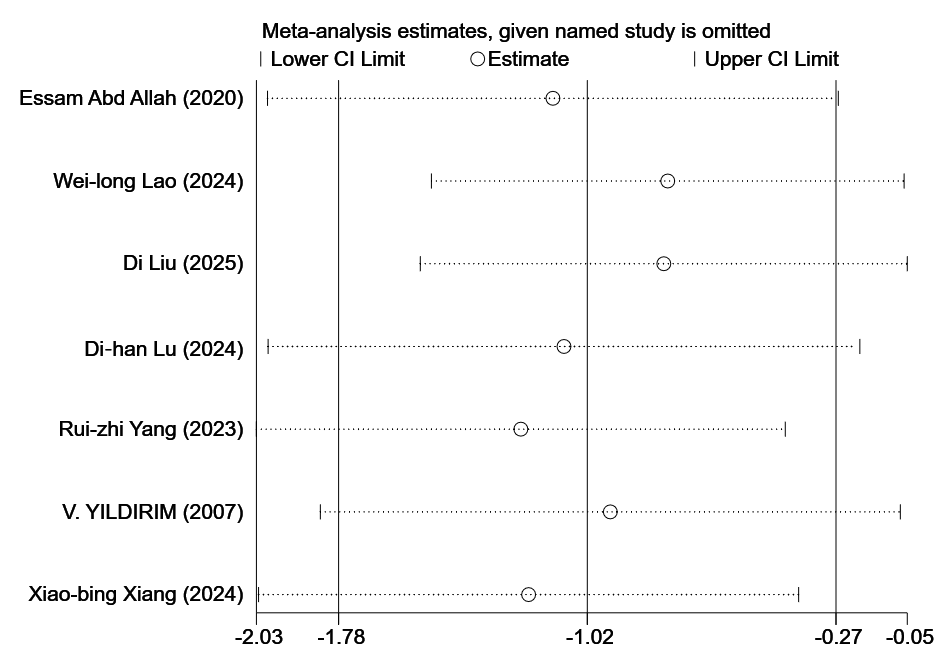


Fig.S8 Sensitivity analysis of hospitalization time


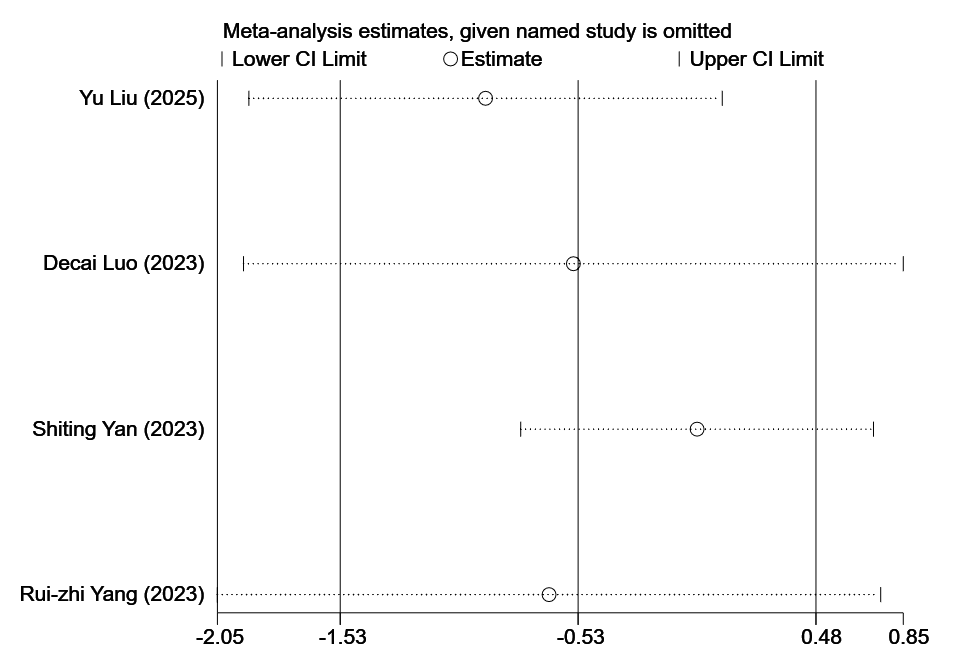


Fig.S9 Sensitivity analysis of sleep quality


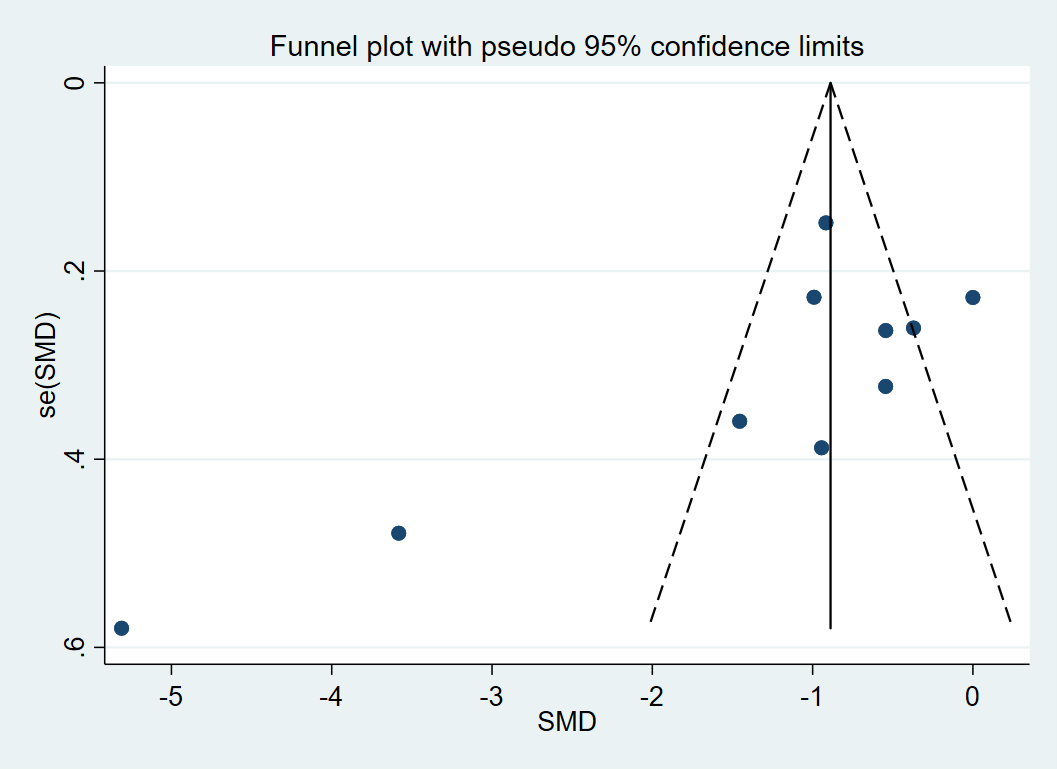


Fig.S10 6-hour pain score funnel plot


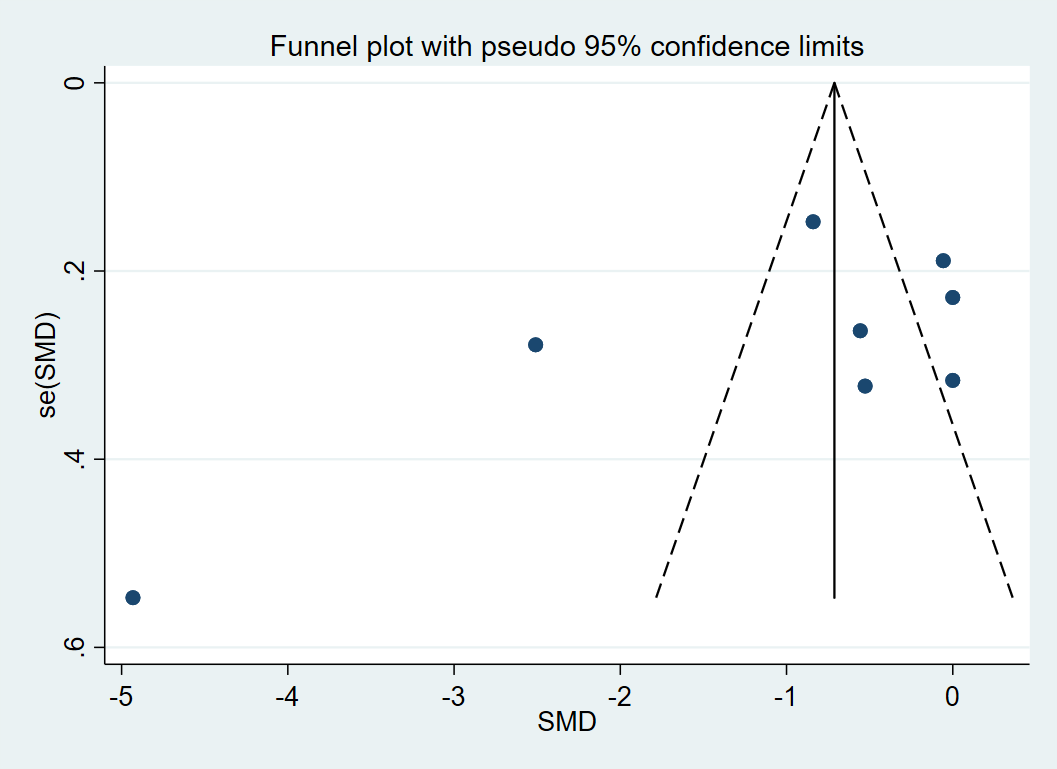


Fig.S11 12-hour pain score funnel plot


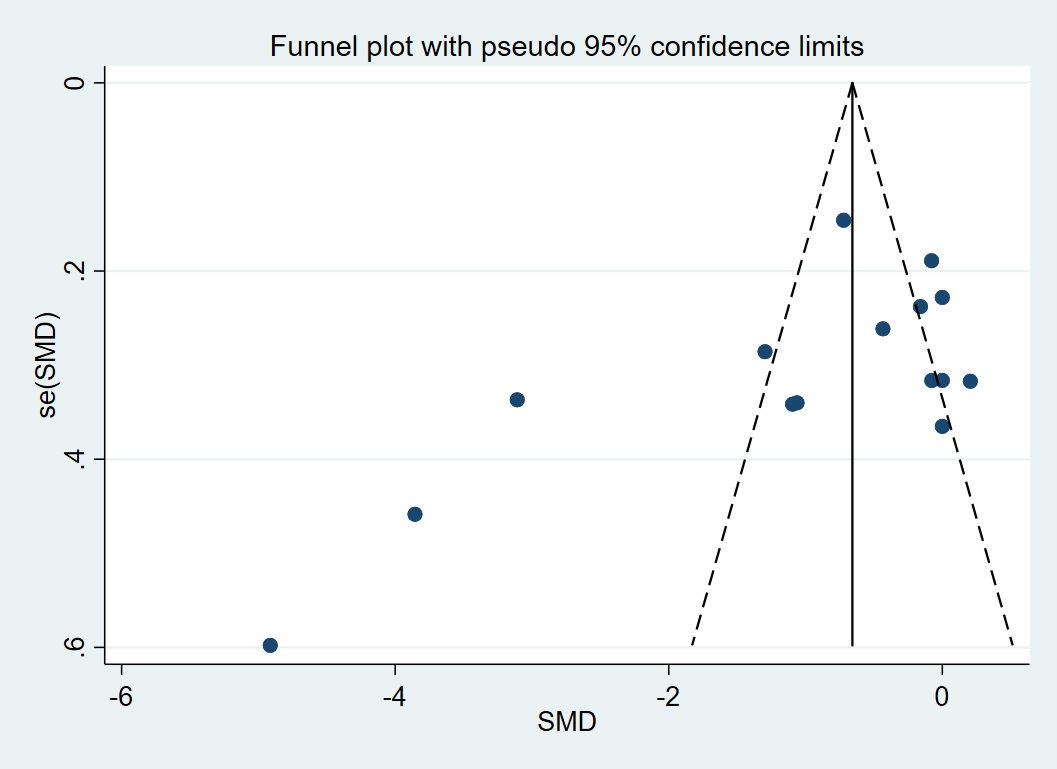


Fig.S12 24-hour pain score funnel plot


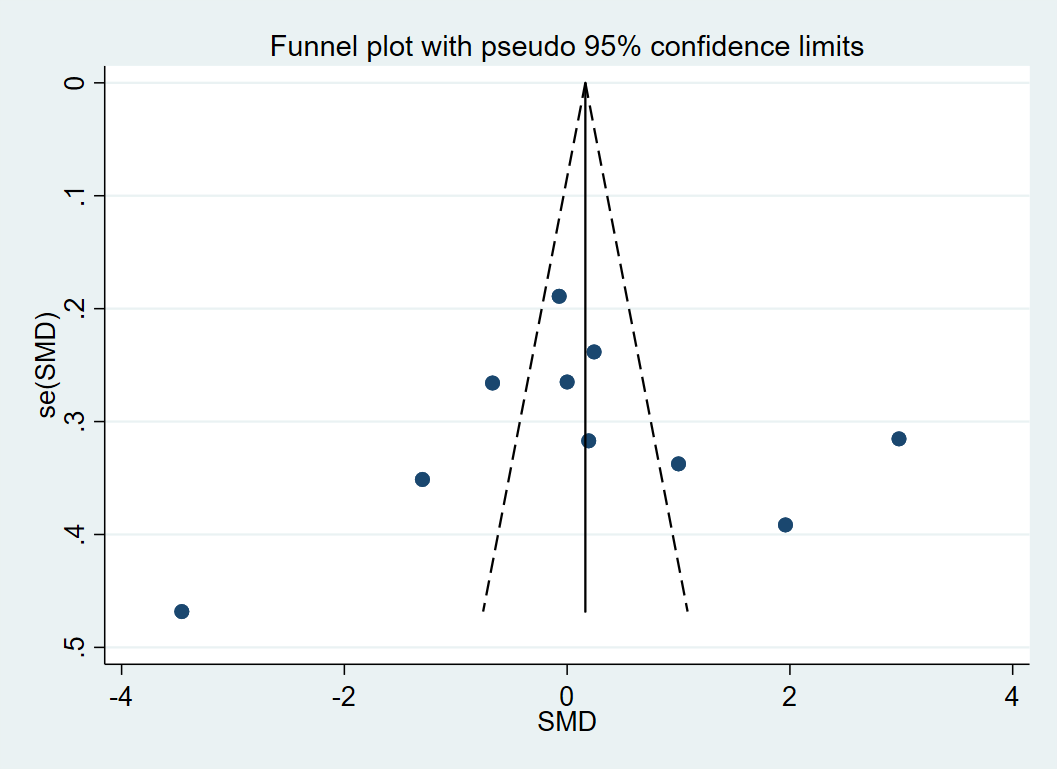


Fig.S13 48-hour pain score funnel plot


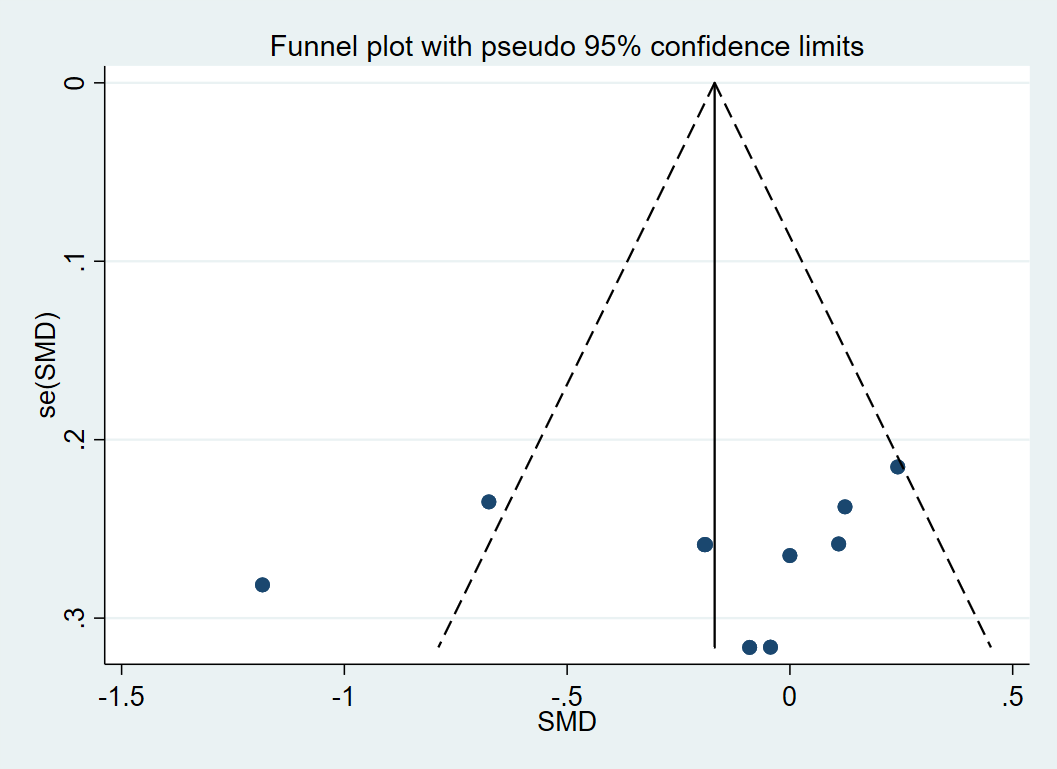


Fig.S14 Funnel plot of total opioid consumption


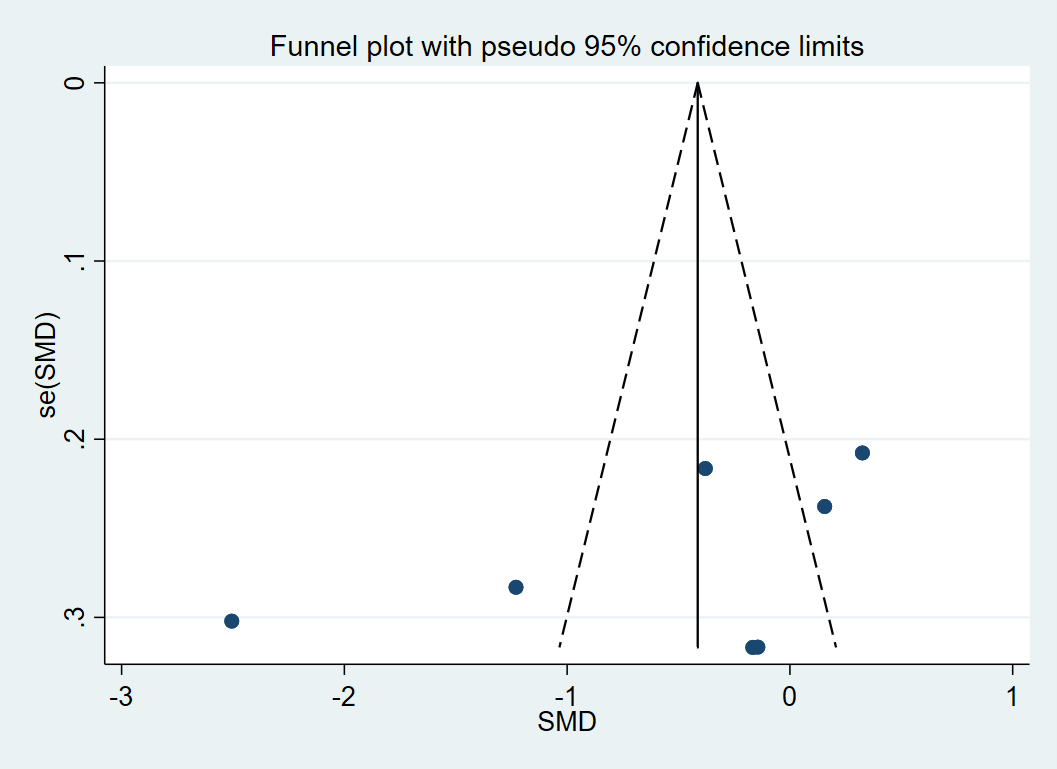


Fig.S15 Funnel plot of total opioid consumption after surgery


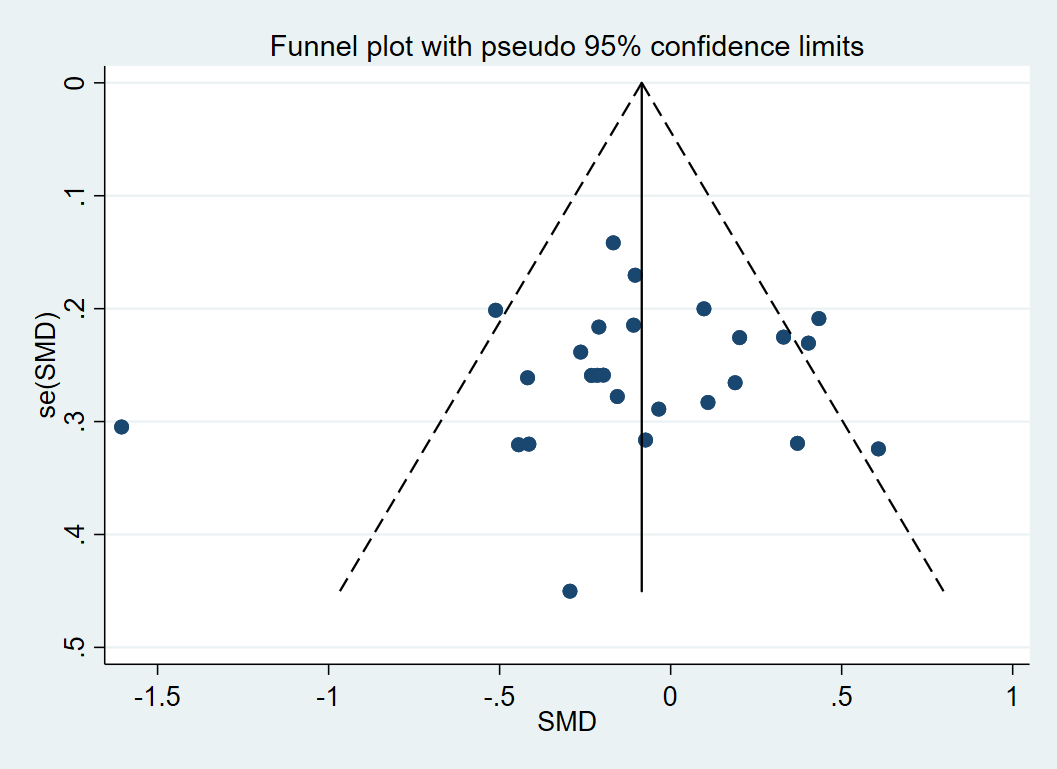


Fig.S16 Funnel plot of surgical operation time


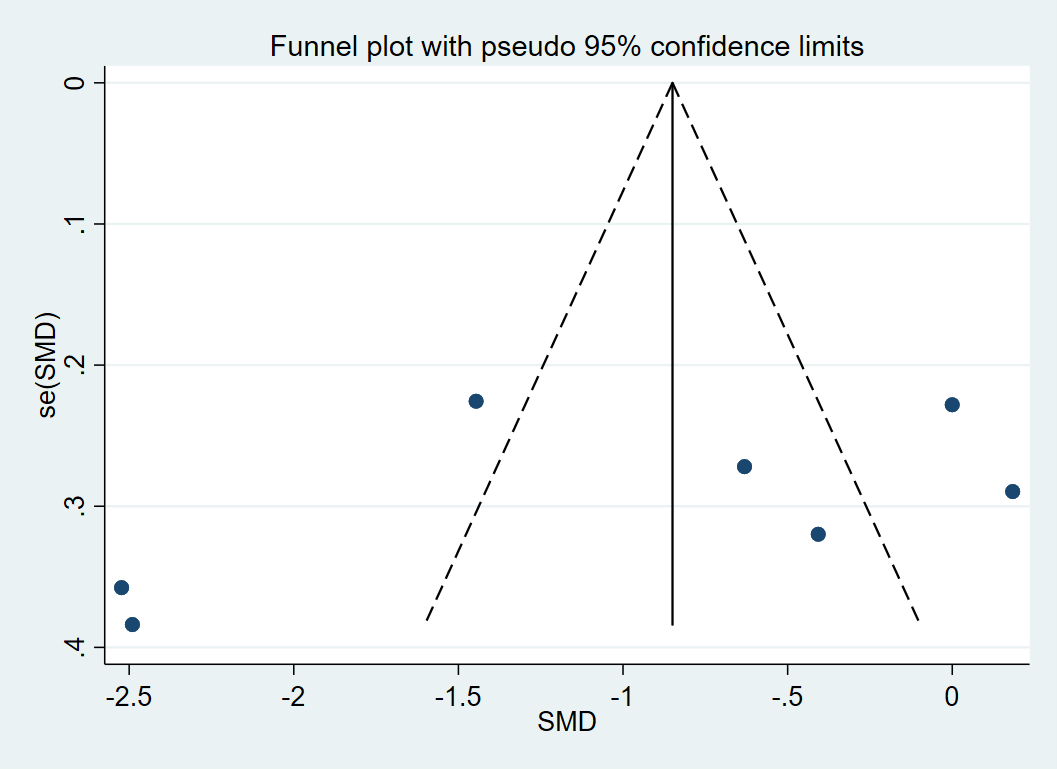


Fig.S17 Funnel plot of hospitalization time


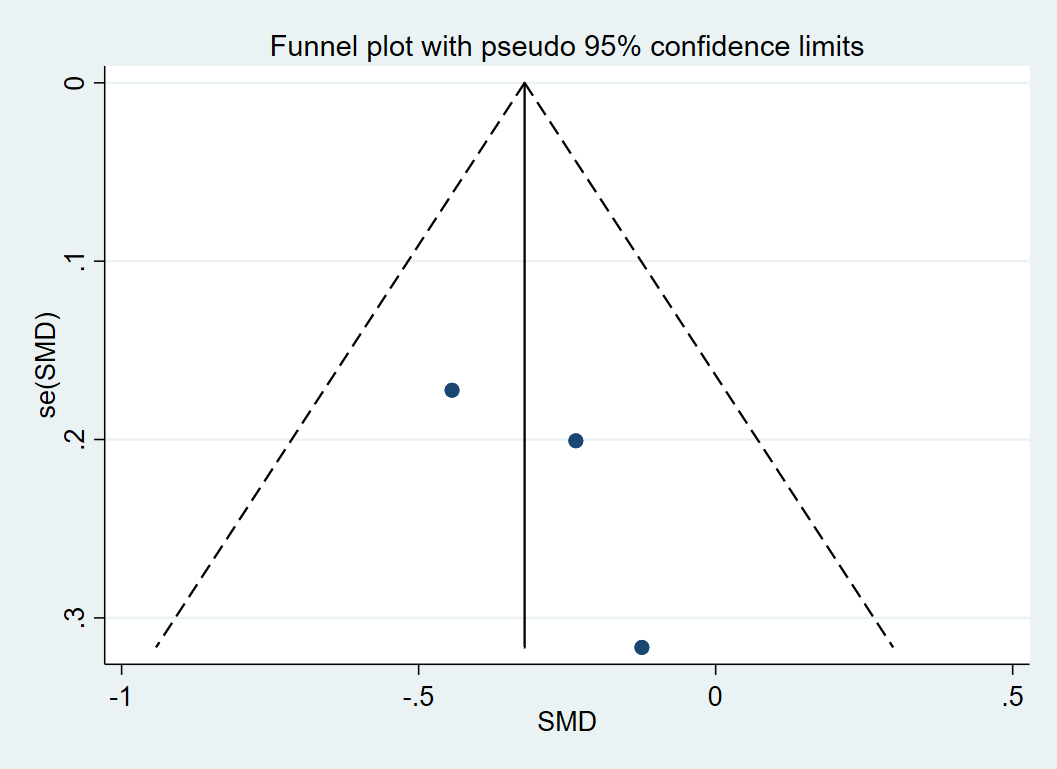


Fig.S18 Funnel plot of ICU stay time


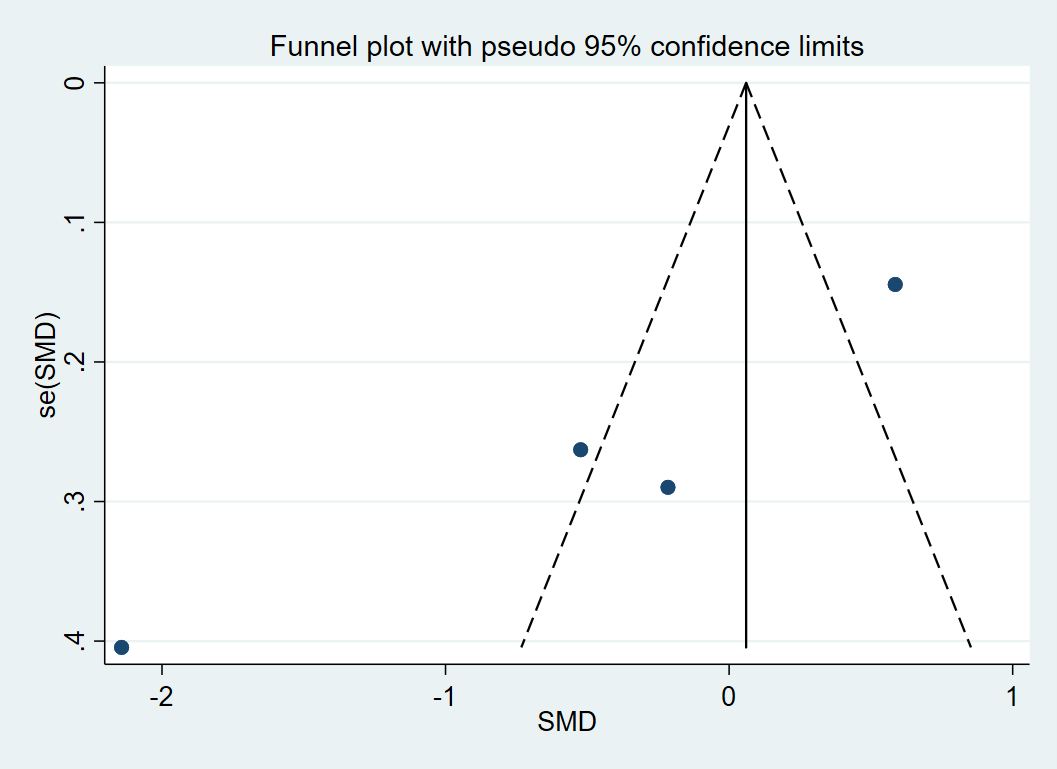


Fig.S19 Funnel plot of sleep quality


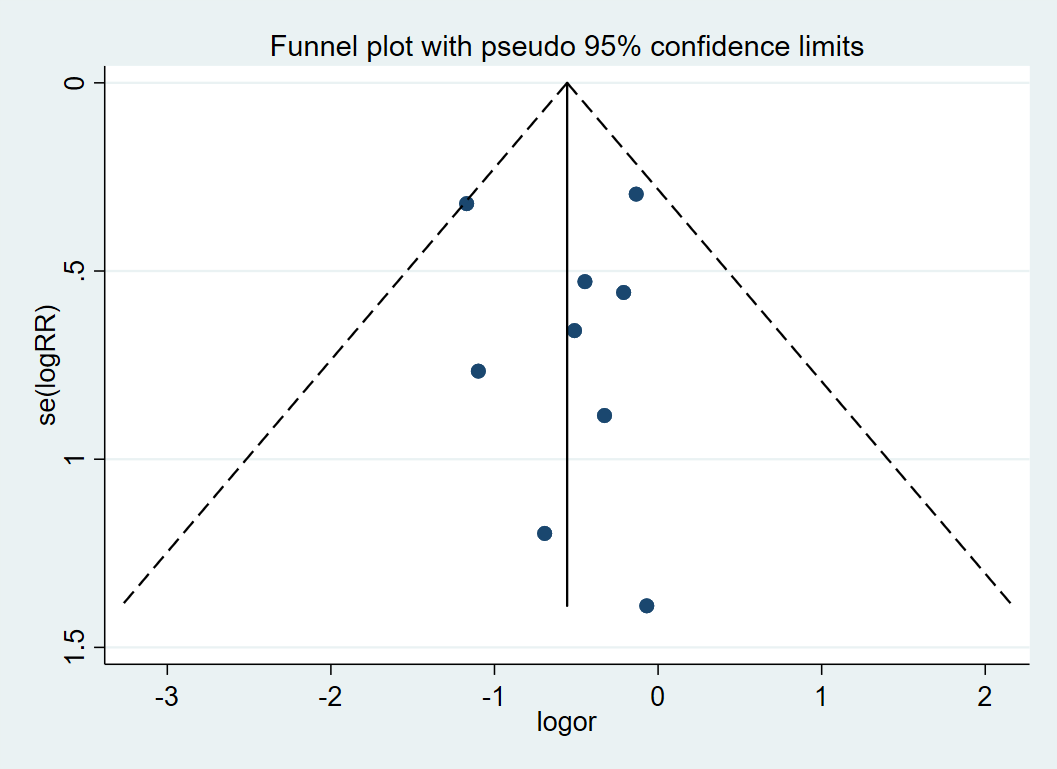


Fig.S20 Funnel plot of nausea and vomiting


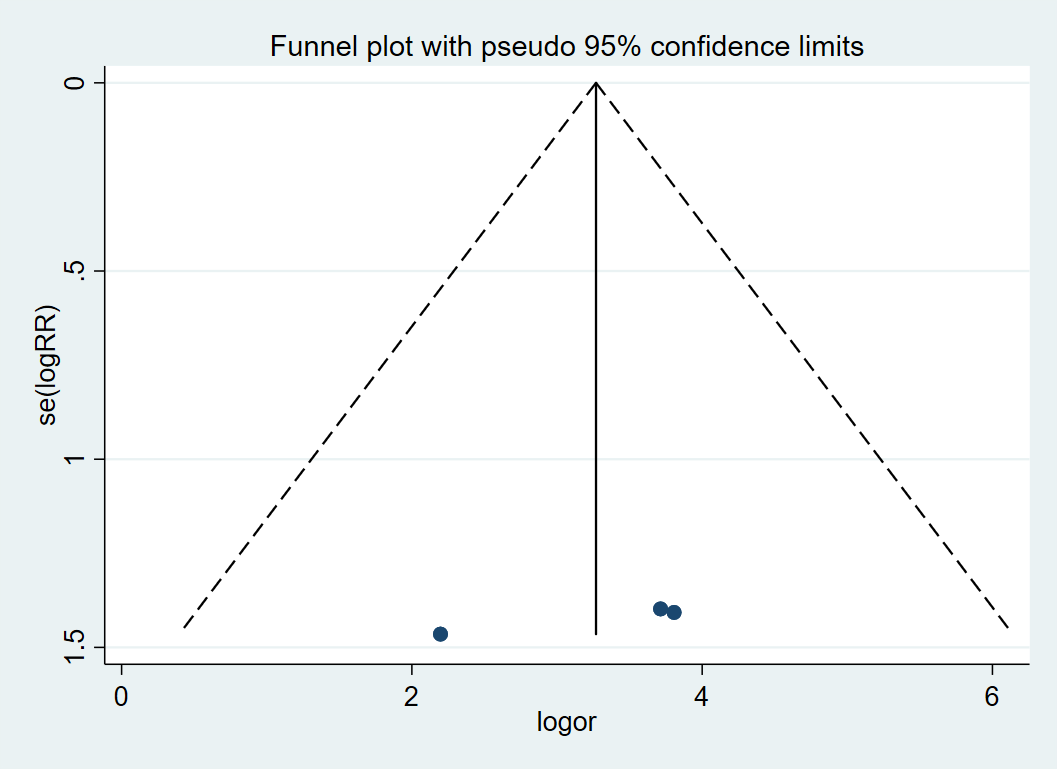


Fig.S21 Funnel plot of upper eyelid ptosis


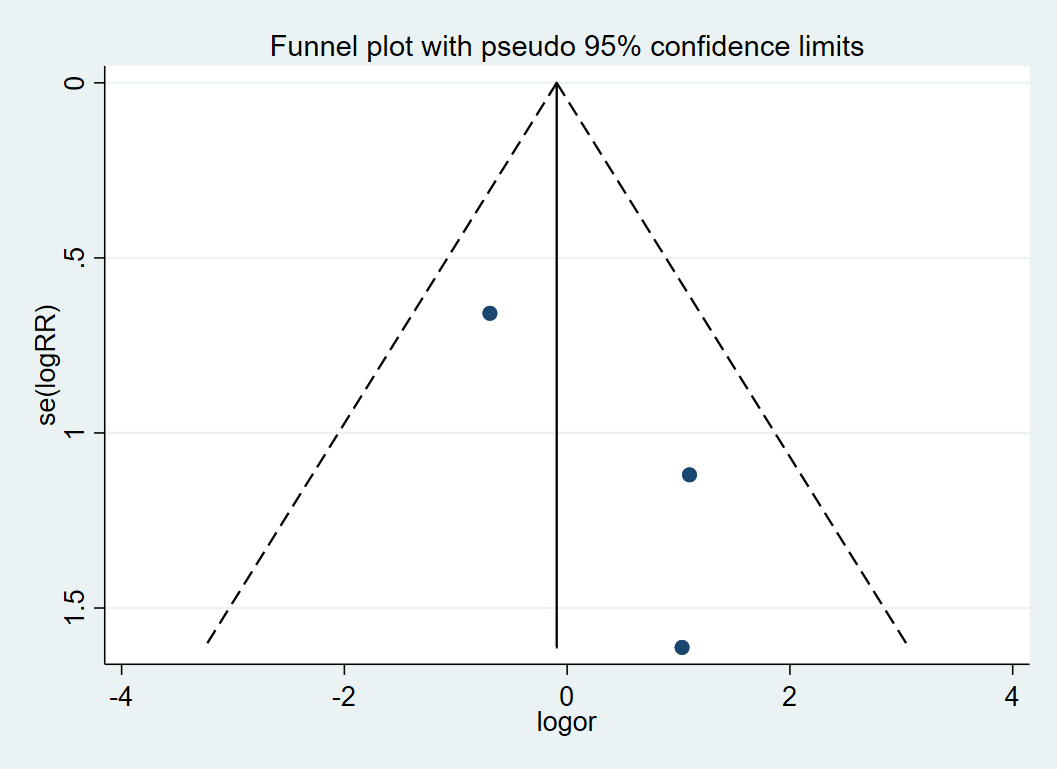


Fig.S22 Infection funnel plot


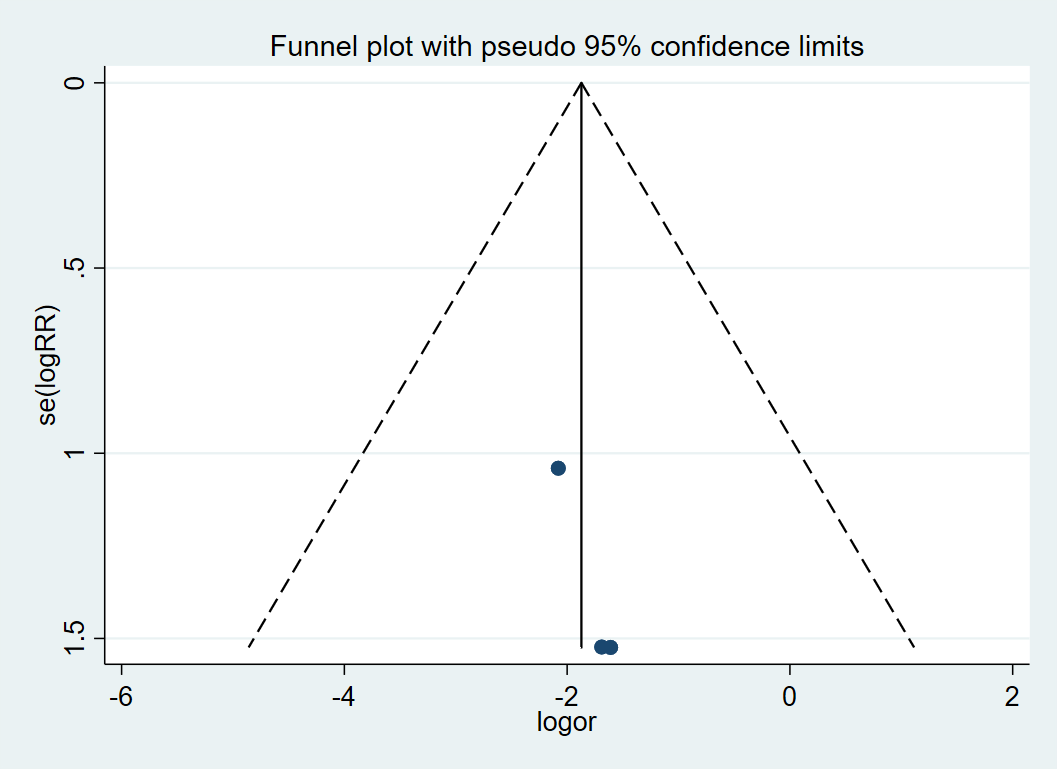


Fig.S23 Dizziness funnel plot
